# Geochemical Influence on Microbial Communities at CO_2_-Leakage Analog Sites

**DOI:** 10.3389/fmicb.2017.02203

**Published:** 2017-11-09

**Authors:** Baknoon Ham, Byoung-Young Choi, Gi-Tak Chae, Matthew F. Kirk, Man Jae Kwon

**Affiliations:** ^1^KU-KIST Green School, Korea University, Seoul, South Korea; ^2^Korea Institute of Geoscience and Mineral Resources, Daejeon, South Korea; ^3^Department of Geology, Kansas State University, Manhattan, KS, United States; ^4^Department of Earth and Environmental Sciences, Korea University, Seoul, South Korea

**Keywords:** CO_2_ leakage analog site, CO_2_ monitoring, groundwater chemistry, bacterial and archaeal community, methanogenesis

## Abstract

Microorganisms influence the chemical and physical properties of subsurface environments and thus represent an important control on the fate and environmental impact of CO_2_ that leaks into aquifers from deep storage reservoirs. How leakage will influence microbial populations over long time scales is largely unknown. This study uses natural analog sites to investigate the long-term impact of CO_2_ leakage from underground storage sites on subsurface biogeochemistry. We considered two sites with elevated CO_2_ levels (sample groups I and II) and one control site with low CO_2_ content (group III). Samples from sites with elevated CO_2_ had pH ranging from 6.2 to 4.5 and samples from the low-CO_2_ control group had pH ranging from 7.3 to 6.2. Solute concentrations were relatively low for samples from the control group and group I but high for samples from group II, reflecting varying degrees of water-rock interaction. Microbial communities were analyzed through clone library and MiSeq sequencing. Each 16S rRNA analysis identified various bacteria, methane-producing archaea, and ammonia-oxidizing archaea. Both bacterial and archaeal diversities were low in groundwater with high CO_2_ content and community compositions between the groups were also clearly different. In group II samples, sequences classified in groups capable of methanogenesis, metal reduction, and nitrate reduction had higher relative abundance in samples with relative high methane, iron, and manganese concentrations and low nitrate levels. Sequences close to *Comamonadaceae* were abundant in group I, while the taxa related to methanogens, *Nitrospirae*, and *Anaerolineaceae* were predominant in group II. Our findings provide insight into subsurface biogeochemical reactions that influence the carbon budget of the system including carbon fixation, carbon trapping, and CO_2_ conversion to methane. The results also suggest that monitoring groundwater microbial community can be a potential tool for tracking CO_2_ leakage from geologic storage sites.

## Introduction

Geologic CO_2_ sequestration is a feasible option for reducing greenhouse gases. Although this strategy is promising, the potential for CO_2_ leakage from the deep subsurface along faults and abandoned wells represents a significant environmental concern (Harvey et al., 2013; Lions et al., 2014). CO_2_ that escapes from deep storage reservoirs to the surface threatens the quality of groundwater resources as well as human populations at the surface. CO_2_ that migrates into an aquifer can decrease water quality by lowering groundwater pH, increasing salinity, dissolving aquifer minerals, and mobilizing hazardous solutes (Harvey et al., 2013; Humez et al., 2014; Lions et al., 2014; Shao et al., 2015).

Coupled with these geochemical impacts, CO_2_ leakage would also influence aquifer microbiology. Previous studies have shown that exposure to high-pressure CO_2_ can adversely affect microorganisms. At pressures of 5–35 MPa, CO_2_ can interfere with cell metabolism through a variety of mechanisms, including cytoplasm acidification, membrane lysis, enzyme deactivation, and trace element mobilization (Watanabe et al., 2003; Bertoloni et al., 2006; Oulé et al., 2006; Wimmer and Zarevúcka, 2010; Santillan et al., 2013; Jin and Kirk, 2016). Nonetheless, studies have also demonstrated that microbial populations can remain active in environments with elevated CO_2_ levels, even in the presence of supercritical CO_2_ (Yakimov et al., 2002; Inagaki et al., 2006; Videmsek et al., 2009; Oppermann et al., 2010; Peet et al., 2015; Emerson et al., 2016; Jin and Kirk, 2016; Probst et al., 2017). Changes in microbial communities following exposure reflect the ability of different species to exist within the perturbed conditions as well as changes in energy available for different microbial reactions (Kirk et al., 2016). Thus, CO_2_ leakage has the potential to affect community composition not only by imposing new stresses on cells but also by altering the redox state of the system.

Most studies that have examined the response of aquifer microorganisms to elevated CO_2_ have considered short time scales (e.g., a few months to at most 3 years). However, CO_2_ leakage could influence subsurface environments for many decades, even up to hundreds of years. Recently, Jin and Kirk (2016) used biogeochemical modeling to examine the response of microbial reactions to leakage over hundreds of years. While data available to constrain their model were limited, the analysis identified potential impacts of CO_2_ leakage on interactions between groups of anaerobes over time that are consistent with some observations from short time-scale studies. Specifically, an increase in CO_2_ decreases energy available for syntrophic oxidation and aceotclastic methanogenesis but increases energy available for microbial iron reduction and hydrogenotrophic sulfate reduction and methanogenesis. Their kinetic model results suggest that high CO_2_ has the potential of promoting iron reduction in ferruginous aquifers, consistent with experimental results obtained by Kirk et al. (2013) and O'Mullan et al. (2015).

Alternatively, the net effects of long-term exposure can be directly examined by analyzing the geochemistry and microbiology of natural analog sites for CO_2_ leakage. For example, several studies investigated the correlation of CO_2_ gradient with microbial community composition, diversity and function in various sites including a geyser (Emerson et al., 2016; Probst et al., 2017), volcanic vents (Beaubien et al., 2008; Oppermann et al., 2010; Fernández-Montiel et al., 2016), and grassland soils near natural CO_2_ springs (Videmsek et al., 2009) and emphasized the relevance of the natural analog sites for CO_2_ leakage from subsurface environments. These studies suggested that elevated CO_2_ in the sites can support a phylogenetically diverse microbial community.

Although these studies have shed light on long-term responses of microbial communities to elevated CO_2_, many questions remain unanswered. In particular, we know little about how the microbial response to high CO_2_ varies under different geochemical conditions, including redox state, salinity, temperature, and the ability of the system to buffer pH. Filling these knowledge gaps is important because feedbacks of the microbial response to CO_2_ exposure have the potential to affect both water quality and CO_2_ trapping (Kirk et al., 2016).

In this study, we investigate the microbial response to high CO_2_ at a natural analog site in South Korea that contains groundwater with variable CO_2_ and major ion chemistry. The objectives of the study are to (1) examine water quality and microbial community compositions at the site, (2) investigate relationships between microbial community composition, CO_2_ exposure, and groundwater geochemistry, and (3) evaluate the potential of groundwater microbial community monitoring as an investigation tool for the fate and transport of CO_2_ leaked from underground storage sites.

## Materials and methods

### Study area and sampling method

**Figure 1:**
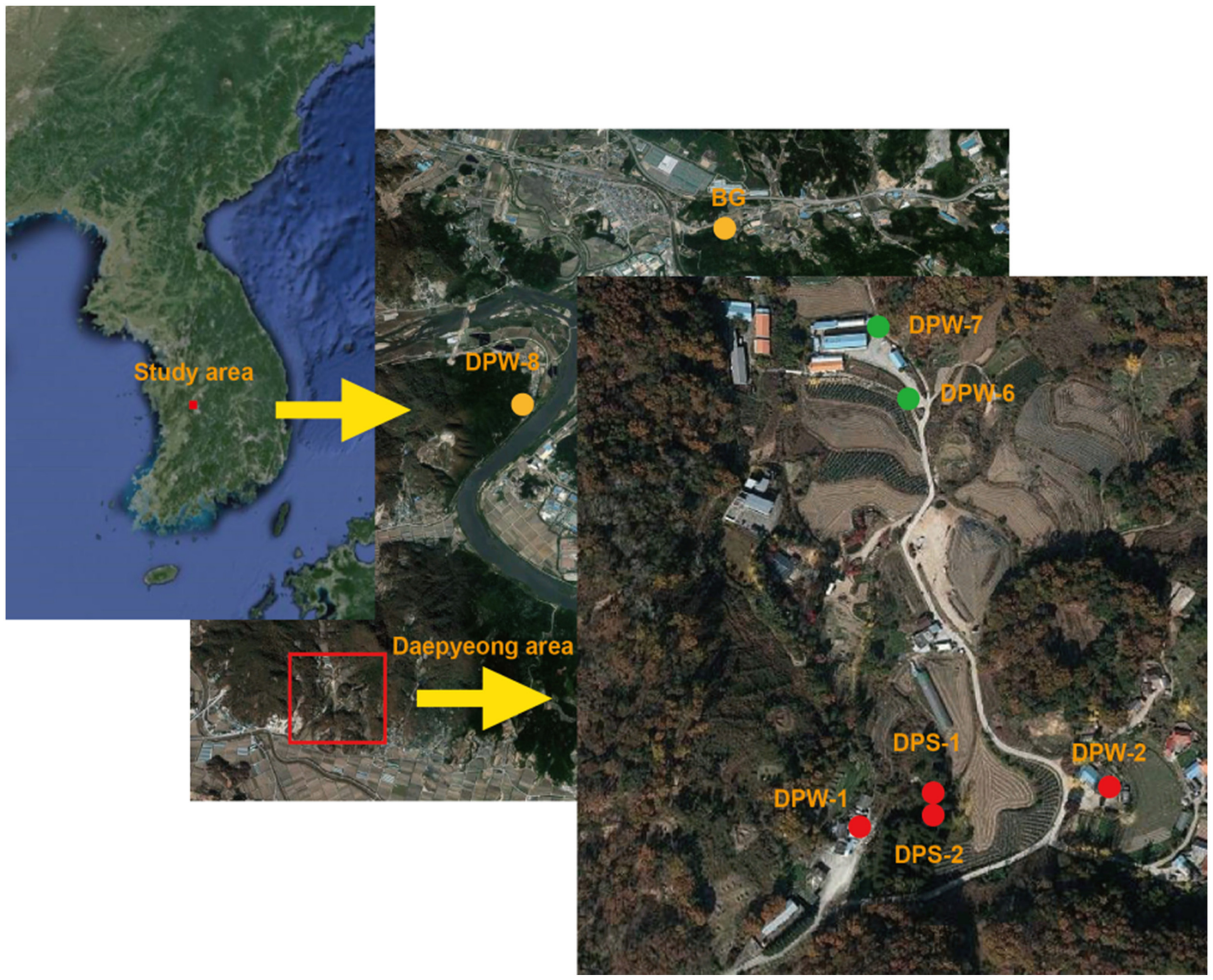
Location map for study area and sampling points in South Korea.

This study area is located about 100 km southeast of Seoul, South Korea (Figure 1). The climate is typical for temperate monsoon regions with an average annual precipitation of 1,225–1,230 mm and more than half (56%) of the annual precipitation falls between June and September (Kim et al., 2008). In addition, monthly mean temperature ranges from 25°C in August to −2°C in January. The geology of the study area mainly consists of Precambrian gneiss and Jurassic biotite granite. The CO_2_-rich waters are located at the geological boundary between Jurassic granites and their adjacent Precambrian gneisses (Jeong et al., 2005; Chae et al., 2013). The granites are mainly composed of K-feldspar, quartz, plagioclase, and biotite (Chae et al., 2013). The origin of CO_2_ gas in this area was known as magmatic CO_2_ gases migrating along faults or fractures (Jeong et al., 2005; Kim et al., 2008).

In this area, the CO_2_-rich waters are produced through several springs and wells. Of them, three springs (DPS-1, DPS-2, and BG) and three wells (DPW-1, DPW-2, and DPW-8) were selected for this study (Figure 1). In addition, two wells (DPW-6 and DPW-7) producing low-CO_2_ groundwater were selected to compare with the CO_2_-rich water and were referred as the low-CO_2_ control group.

Water samples for this study were collected in January and April 2014. For geochemical analyses, the spring water samples were collected using a peristaltic pump with care taken to minimize formation of gas bubbles resulting from CO_2_ degassing. Before groundwater sampling, the wells were purged until temperature and electrical conductivity (EC) of the water stabilized. All water samples were filtered using 0.45 μm membrane filter paper. The filtrate for the analysis of cations (Na^+^, K^+^, Ca^2+^, Mg^2+^, and dissolved Fe) and silica was acidified with nitric acid and that for the analysis of anions (F^−^, Cl^−^, Br^−^, ${\text{NO}}_{3}^{-}$, ${\text{SO}}_{4}^{2-}$) was unacidified. All samples were stored in pre-cleaned high-density polyethylene (HDPE) bottles at 4°C until laboratory analyses.

Sampling for dissolved gases (CO_2_ and CH_4_) targeted the CO_2_-rich waters only. The CO_2_ water was pumped through Tygon tubing into a HDPE container. Water from the peristaltic pump outflow was used to fill a large bucket, the Tedlar gas sampling bag (1 L) was immersed in the bucket and then connected with the tube. The sampling bag was filled with about 500 mL of water without gas bubbles and then the tube was removed. The collected samples were also stored at 4°C until laboratory analyses.

### Chemicals

All chemicals used were of reagent grade quality or higher. Distilled deionized water (ddH_2_O) prepared with a Millipore water purification system (Barnstead, USA) was used throughout. All the analytical procedures have been validated using certified and/or internal reference materials.

### Physical and chemical analyses

Field parameters, such as pH, Eh, EC, and dissolved oxygen (DO) were measured in the field using electrodes avoiding contact of water with air. Alkalinity was also determined in the field by acid titration with 0.05 N HNO_3_ and reported as ${\text{HCO}}_{3}^{-}$ (mg L^−1^). Concentration of dissolved organic carbon (DOC) was determined by the total organic carbon analyzer (Shimadzu, TOC-L CPH, Japan). The concentrations of major cations were measured by inductively coupled plasma-optical emission spectrometry (ICP-OES, Varian 730-ES, USA) and those of anions (Br, Cl, F, NO_3_, and SO_4_) were determined by ion chromatography (Metrohm 850 professional IC, Switzerland). Trace elements were determined by inductively coupled plasma mass spectrometer (ICP-MS, ELAN DRC-e) (Perkin-Elmer SCIEX Instruments, Concord, Canada). The concentrations of CO_2_ and CH_4_ were also analyzed using gas chromatography (GC, Agilent 6890) (Agilent Technologies Inc., Santa Clara, CA, USA). pCO_2(g)_ of group II samples was calculated using PHREEQC based on the measured values of pH (H^+^) and alkalinity (${\text{HCO}}_{3}^{-}$). Careful quality control and analysis of data was conducted by measuring blanks, duplicates, and standards. The charge balance errors for the analyzed data were generally below ±5%.

### Microbial community analysis

#### Biomass collection

For microbial analyses, about 20 L of water was passed through a 0.2 μm membrane filter paper (Pall Gelman Laboratory, Ann Arbor, MI) using stainless filter holder (Millipore Corp.) for 30 min, which was connected to the peristaltic pump in the springs or to the discharge ports in the wells. After filtration the filter papers were stored in a 50 mL conical tube and then stored at −20°C in the lab. All equipment was sterilized in an autoclave before sampling.

#### DNA extraction

Samples were frozen at −20°C until the experiments were completed. The filters were cut into several small pieces and dispensed into a sterile microcentrifuge tube. Total genomic DNA was extracted by using an i-geonomic Soil DNA Extraction Mini Kit (iNtRON, South Korea) with a bead-beating apparatus, according to the manufacturer's directions. DNA concentration was quantified with a Qubit fluorometer (Invitrogen, USA), by following the manufacturer's instructions. The extracted DNA was used for PCR amplification for clone library and MiSeq sequencing as described below.

#### Clone library and high-throughput sequencing

For PCR amplification, the DNA template (100 ng) was added to a master mix of ddH_2_O, 2.5 μL of 10 × PCR buffer, 1 μL of 50 mM MgSO_4_, 0.5 μL of 10 mM dNTPs, 2 μL of 8F primer (5′-AGAGTTTGATCMTGGCTCAG-3′)(10 μM), 2 μL of 1492R primer (5′-TACGGYTACCTTGTTACGACTT-3′)(10 μM), and 0.2 μL of *i-Taq* DNA polymerase (5 U μL^−1^, Intron, South Korea), and brought to a final volume of 25 μL with sterile ddH_2_O. For amplification of archaeal DNA, primers 109F (5′-ACKGCTCAGTAACACGT-3′) and 958R (5′-YCCGGCGTTGAMTCCAATT-3′) from the same source were used. Templates were amplified by using a thermal cycler. The initial denaturation step at 94°C for 2 min was followed by 30 cycles of 94°C (30 s), 58°C (45 s), and 72°C (90 s), with a final extension at 72°C for 10 min. The PCR products were purified as indicated above. Clone libraries were constructed by using the T-Blunt™ PCR Cloning Kit (with DH5α Competent *Escherichia coli*; Solgent, South Korea). Approximately 50 colonies per plate were selected randomly and re-amplified by using the vector-specific primers (0.25 μM each) M13F (5′-GTAAAACGACGGCCAG-3′) and M13R (5′-CAGGAAACAGCTATGAC-3′) (10 μM, BIONEER, Korea). M13 primer-amplified PCR products were visualized on a 1% agarose gel (Invitrogen) to check for successful amplification of the cloned inserts. The successful products were then treated with ExoSAP-IT (USB, CA, USA) (1.5 μL of product, 3.25 μL of ddH2O, and 0.25 μL of ExoSAP-IT for each reaction). Purified M13 PCR products were sequenced at the Marcrogen (Seoul, Korea) with the 8F forward primer.

#### Sequence analysis of clone library

All sequences were trimmed by using DNA Baser (Heracle BioSoft, Romania) to remove inconclusive nucleotides from the ends, on the basis of the number of ambiguous base calls and quality scores. The sequences were also screened for chimeras and other artifacts with the Mallard program (http://www.cardiff.ac.uk/biosi/research/biosoft/) (Ashelford et al., 2006). A multiple-sequence alignment prepared for each included the sequence *E. coli* U00096 as a reference. The sequences screened were analyzed vs. publicly available bacterial 16S rRNA gene sequences from the Ribosomal Database Project (RDP) Release 11.5 consists of 3,356,809 aligned and annotated 16S rRNA sequences through the RDP-II website (http://rdp.cme.msu.edu/). Distance matrices based on alignment of the library sequences to the RDP database were used as input for further analysis with the Distance Based Operational Taxonomic Units (OTUs) and Richness Determination (DOTUR) program (Schloss and Handelsman, 2005). All clones having a mutual sequence similarity of more than 97% were grouped into OTUs by using DOTUR. Rarefaction curves (number of OTUs detected vs. the number of clones analyzed) were calculated to explore the diversity of microbial communities.

#### MiSeq sequencing

We used two sets of primer pairs for MiSeq library construction. Taxonomic coverage of two forward primers and two reverse primers was evaluated by probe match tool of the Ribosomal Database Project (RDP) according to previous studies (Cole et al., 2014; Hugerth et al., 2014). For the first library, we used the primers 515F (5′-GTGCCAGCMGCCGCGGTAA-3′) and 805R′, (5′-TACHVGGGTATCTAATCC-3′) (Caporaso et al., 2011) targeting hypervariable region 4 (V4) of the 16S rRNA genes of bacteria and archaea. The PCR conditions were 94°C for 3 min, followed by 30 cycles of 94°C for 30 s, 50°C for 60 s, and 72°C for 90 s, followed by a final extension at 72°C for 10 min. For the second library construction, the DNA extracted from groundwater samples were amplified with 341F (5′-CCTACGGGNGGCWGCAG-3′) and 805R (5′-GACTACHVGGGTATCTAATCC-3′) set of primers (Klindworth et al., 2012) targeting the V3–V4 of 16S rRNA gene. The PCR conditions were 95°C for 5 min, followed by 30 cycles of 95°C for 30 s, 55°C for 30 s, and 72°C for 30 s, followed by a final extension at 72°C for 5 min. Each sample was amplified in three replicate 25-μL PCR reactions and then purified using the UltraClean PCR clean-up kit (Mo Bio Laboratories, Solana Beach, CA, USA) and combined into a single volume. Purified DNAs were quantified by incorporating Picogreen (Invitrogen) according to the manufacturer's instructions. 16S rRNA-based MiSeq sequencing with a paired-end read length of 2 × 300 bp was conducted at Macrogen (Seoul, Republic of Korea) using the Illumina MiSeq system (Illumina, USA), according to the manufacturer's instructions.

#### Sequence analysis of MiSeq

To perform quality filter and merge simultaneously, we used the Illumina Utilities library as described previously in Eren et al. (2013) with an enforced Q30-check and ≤3 mismatches required to retain a sequence and its source code is available from (https://github.com/meren/illumina-utils). Sequencing data were analyzed using QIIME 1.8.0 (Caporaso et al., 2010). The detailed QIIME parameters used in the current analysis are following: The “pick_otus.py” script was used to cluster reads from all samples into OTUs based on nucleotide sequence identities. The clustering method was “Uclust” (Edgar, 2010). As default parameters, the similarity threshold was set at 0.97 (roughly corresponding to species-level OTUs). After picking OTUs, the “pick_rep_set.py” script was used for picking a representative set of sequences. For the best taxonomic assignment of the sequences, we used two taxonomy reference databases, RDP and SILVA. Both databases showed similar results, but SILVA database showed better taxonomic assignment particularly for Archaea (Table S1). The “assign_taxonomy.py” was used to assign the taxonomy of each sequence, with the 24 July 2014 release of the ARB-formatted SILVA small subunit (SSU) reference database (SSU Ref v.119) downloaded from www.arb-silva.de/documentation/release-119 (Quast et al., 2012). The “align_seqs.py” was used to align with the SILVA 119 database, and the template file used was SILVA 119 reference “core_Silva119_alignment.fna.” To filter out chimeric sequences after the alignment “filter_alignment.py” script was used with the template (lanemask_in_1s_and_0s). The “make_phylogeny.py” script was used with the “FastTree” program (Price et al., 2010) to construct a phylogenetic tree. The “make_otu_table.py” script was used to generate an OTU table in the biom format. The “filer_otu_from_otu_table.py” script was used to remove all OTUs that were observed fewer than two times (i.e., singletons). The “filter_taxa_from_otu_table.py” script was used to remove Archaeal taxa from an OTU table.

To calculate species diversity and richness within individual samples, alpha diversity analysis, including observed OTUs, Chao1 (Chao, 1984), Shannon (Shannon, 2001), Simpson (Simpson, 1949), ACE (Chazdon et al., 1998) indices for statistical tests, was processed using the QIIME script. Good's coverage estimator was also used to calculate the sequence coverage obtained for the 16S rRNA datasets (Good, 1953). To measure the similarity among communities, beta diversity was calculated based on weighted and unweighted unifrac distance matrices between communities and then 3D principle coordinates analysis (PCoA) plots were constructed. Non-metric multidimensional scaling (NMDS) analyses were performed with the function “meta.mds()” of the package vegan implemented in R (R Core Team, 2014), based on the microbial community information (relative abundance of each OTU detected by either MiSeq sequencing or clone library). The vector fitting of environmental variables to the NMDS ordination was determined by the “envfit()” function in the vegan package with 16 major components of physical and chemical characteristics. Significance was determined based on Bray–Curtis distances and 10,000 random permutations. Diversity metrics were calculated based on the sequencing data analyzed by 341-805R, with the minimum number of reads being 30,714 for bacteria and 1,974 for Archaea (Tables S1, S2).

#### Accession codes

Sequencing reads produced from Miseq during this study have been deposited at the NCBI Short Read Archive(SRA) under project accession number SRP119027 (Accession number: SRR6110859-SRR6110865).

## Results and discussion

### Geochemical characteristics

The hydrochemical data collected in January and April 2014 are presented in Table 1. The data between two sampling events were almost identical except water temperature at DPS-2. Consistent with our expectations, water samples from groups I and II (i.e., the high-CO_2_ groups) had higher CO_2_ levels than those from group III. Dissolved CO_2_ concentrations, calculated based on our water chemistry results, were about 28 times higher for groups I and II compared to group III, on average. Reflecting these differences in CO_2_ levels, samples from group I and II ranged to lower pH levels than those from group III. pH ranged from 4.5 to 4.8 for group I samples, 6 and 6.2 for group II samples, and 6.2 and 7.3 for group III.

**Table 1:** Physical and chemical characteristics of ground water samples.

**Name**	**Group I**				**Group III**		**Group II**	
	**DPS**	**DPS**	**DPW**	**DPW**	**DPW**	**DPW**	**DPW**	**BG**
	−1^*^	**−2**	**−1**	**−2**	**−6**	**−7**	**−8**	
Sampling depth (m)	0	0	100	100	100	100	700–800	0
**WATER QUALITY PARAMETERS**								
T (°C)	4.9	9 ± 2.6	13.8 ± 0.1	13.5 ± 0.2	15.9 ± 0.3	14.7 ± 0.1	24.4 ± 0.3	13.6 ± 0.6
pH	4.8	4.8 ± 0	4.7 ± 0.3	4.5 ± 0	7.3 ± 0.2	6.2 ± 0	6.2 ± 0	6 ± 0
EC (μS cm^−1^)	136	188 ± 15	152 ± 2	160 ± 5	219 ± 3	188 ± 3	2,095 ± 17	1,565 ± 4
TDS (mg L^−1^)	86.3	109.9 ± 14.6	106.5 ± 8.7	93.0 ± 10.9	94.4 ± 4.5	103.6 ± 4.7	650.8 ± 34.2	475.9 ± 4.1
DO (mg L^−1^)	2.1	1.9 ± 0.3	1.1 ± 0.2	0.8 ± 0.2	2.6 ± 0.1	2.3 ± 0.4	0.8 ± 0.6	1.0 ± 0.1
Eh (mV)	216.6	183.8 ± 9.2	280.8 ± 37.2	311.9 ± 33.2	120.4 ± 20.9	217.1 ± 64.4	59.0 ± 22.8	105.0 ± 18.6
Alkalinity_†_ (mg L^−1^)	23.9	26.9 ± 5.2	18.7 ± 0.8	34.7 ± 7	86.7 ± 1.4	68.7 ± 3.3	1,395.3 ± 2.5	1,067.3 ± 0.3
DOC^*^ (mg L^−1^)	1.7	1.1	0.6	2.3	0.7	1.5	1.7	4.7
**MAJOR IONS (mg L^−1^)**								
Na^+^	11.7	12.9 ± 0.6	12.5 ± 0.5	10.6 ± 0.5	12.8 ± 0.3	12.2 ± 0.1	245.1 ± 8.6	63.1 ± 7.7
K^+^	1.5	2.1 ± 0.2	2.1 ± 0.1	2 ± 0.2	1.1 ± 0.1	1.1 ± 0.1	7.5 ± 0.5	2.6 ± 0.1
Ca^2+^	10.1	17 ± 2.5	12.5 ± 0.3	12.4 ± 1.1	32.7 ± 0.6	22.2 ± 0.1	250.1 ± 8.2	293 ± 3.6
Mg^2+^	2.5	3.3 ± 0.1	3 ± 0.1	2.9 ± 0.3	2.6 ± 0.1	4.7 ± 0.1	22.9 ± 0.0	16.4 ± 0.2
SiO_2_	36.2	48.3 ± 6.3	51.7 ± 7.1	48.2 ± 8	26.3 ± 3	39.3 ± 4.4	88.2 ± 9.4	74.4 ± 5.4
F^−^	0.3	0.2 ± 0.1	0.3 ± 0.1	0.3 ± 0.1	1.0 ± 0.0	0.2 ± 0.0	2.0 ± 0.5	1.6 ± 0.4
Cl^−^	10.2	10 ± 1.2	9.2 ± 0.3	5 ± 0.2	4.8 ± 0.2	6.9 ± 0	9.9 ± 0.7	2.2 ± 1.2
Br^−^	0.9	1.4 ± 0.5	1.7 ± 0.4	2.2 ± 0.5	<0.1	<0.1	2.7 ± 0.1	2.7 ± 0.3
${\text{NO}}_{3}^{-}$	4.7	11.3 ± 2.6	9.8 ± 0.5	7.6 ± 0.5	6.4 ± 0.5	6.4 ± 0.5	0.1 ± 0.1	0.1 ± 0.1
${\text{SO}}_{4}^{2-}$	8.2	3.7 ± 0.9	3.8 ± 0.3	2.1 ± 0	7 ± 0	10.7 ± 0.3	10.3 ± 0.7	8.7 ± 0.3
Fe	<0.1	<0.1	<0.1	<0.1	<0.1	<0.1	5.3 ± 0.6	11.3 ± 1.7
**MINOR IONS (μg L^−1^)**								
Mn	64.7	74.1 ± 2.7	72.4 ± 2.6	59.2 ± 7.7	0.4 ± 0.0	0.2 ± 0.0	616.6 ± 17.9	990.3 ± 70.8
Sr	78.3	121.3 ± 4.8	112.8 ± 6.9	103.4 ± 12.5	111.5 ± 11.8	138.2 ± 11.9	1, 717.2 ± 10.2	1, 485.5 ± 33.4
Al	171.4	166.1 ± 13.0	170.5 ± 19.6	282.3 ± 54.7	<5.0	<5.0	20.3 ± 0.7	72.6 ± 8.4
Li	7.2	10.6 ± 0.6	11.9 ± 0.9	13.0 ± 1.8	13.0 ± 0.9	10.9 ± 0.6	1, 271.1 ± 217.0	448.9 ± 46.8
Be	0.5	0.9 ± 0.1	0.9 ± 0.1	0.8 ± 0.1	<0.1	0.1 ± 0.0	28.8 ± 2.4	23.6 ± 1.7
Cr	1.8	4.2 ± 0.2	3.4 ± 0.0	6.5 ± 0.9	<0.5	<0.5	18.0 ± 0.6	13.6 ± 0.6
Ni	1.1	1.6 ± 0.3	1.4 ± 0.1	1.8 ± 0.4	0.6 ± 0.2	0.5 ± 0.1	3.7 ± 0.2	4.3 ± 0.5
Zn	5.2	9.5 ± 0.8	8.2 ± 0.4	7.5 ± 0.9	8 ± 0.8	3.1 ± 0.1	222.4 ± 32.9	0.5 ± 0.2
Ga	0.4	0.7 ± 0.1	0.5 ± 0.0	0.7 ± 0.1	<0.1	<0.1	0.6 ± 0.1	0.8 ± 0.1
Rb	4.7	6.7 ± 0.6	7.4 ± 0.5	7.6 ± 0.6	0.2 ± 0.1	0.2 ± 0.1	51.8 ± 0.1	15.3 ± 0.3
Cs	0.1	0.2 ± 0.1	0.2 ± 0.0	0.3 ± 0.0	<0.1	<0.1	16.1 ± 0.8	2.4 ± 0.1
Ba	14.5	23.2 ± 0.5	19.0 ± 0.9	24.1 ± 1.9	0.3 ± 0.0	1.4 ± 0.4	26.0 ± 1.3	30.2 ± 1.1
**GAS CONCENTRATION**								
CH_4_(g)^*^ (mg L^−1^)	nd^a^	nd	nd	nd	ND^b^	ND	35.0	22.4
CO_2_(g)^*^ (mg L^−1^)	2, 152.7	2,404.1	2,116.6	3,223.2	ND	ND	3,375.8	3,889.5
PCO_2_(g) (Logarithmic scale)	−0.5	−0.39 ± 0.02	−0.19 ± 0.26	−0.06 ± 0.03	−2.51 ± 0.15	−1.35 ± 0.02	−0.06 ± 0.03	−0.06 ± 0.01
**SATURATION INDEX (SI)**								
Calcite	−4.34	−4.19 ± 0.04	−4.40 ± 0.35	−4.33 ± 0.17	−0.80 ± 0.24	−2.17 ± 0.03	0.08 ± 0.01	−0.32 ± 0.04
Dolomite	−7.98	−7.78 ± 0.13	−8.08 ± 0.70	−7.95 ± 0.35	−1.35 ± 0.48	−3.68 ± 0.08	0.53 ± 0.04	−0.54 ± 0.06
Siderite	nc^c^	nc	nc	nc	nc	nc	0.20 ± 0.04	0.12 ± 0.12

a *nd, not detected*,

b *ND, not determined*,

c *nc, not calculated because of no dissolved iron in water*,

* *Collected only in January 2014, † as ${\text{HCO}}_{\text{3}}^{-}$, Mean values (±standard deviation) of water quality parameters and ion concentrations are provided for sites sampled in both January and April. DOC and gas concentrations were determined only in January. DPS1 data were collected only in January*.

The distributions of major ion concentrations between groups are shown in Table 1. Group I and group III show the similar ranges of ion concentrations and have considerably lower concentrations of SiO_2_, Ca^2+^, Mg^2+^, Na^+^, K^+^, and HCO^3−^ than group II (Table 1). These ions commonly indicate the extent of water-rock interactions because they are released during dissolution of silicate and carbonate minerals. Thus, our results suggest that water samples in group I and group III have undergone less water-rock interaction than those in group II.

High levels of CO_2_ are favorable for mineral dissolution as acid produced by CO_2_ dissolution reacts with carbonate and silicate minerals (Kharaka et al., 2006; Oelkers et al., 2008; Raistrick et al., 2009). As such, it may be expected that groups I and II would likely both show extensive water-rock interaction. However, a previous work by Kim et al. (2008) indicates that differences between the two reflect the residence time of groundwater in the aquifer. Groundwater from group I appears to be relatively recent recharge (the apparent groundwater age was 32 years) (Chae et al., 2013) that has had CO_2_ added to it whereas groundwater from group II resulted from the long-term water-rock interactions after the influx of CO_2_ gases.

Samples from each group also differ in terms of redox state. Measured Eh values indicate that samples from group II came from a more reduced environment than those from group I and III (Table 1). Consistent with those values, samples from groups I and III had higher DO and nitrate levels than those from group II. Moreover, methane and dissolved iron were above detection limits only for group II samples.

### Microbial community dynamics

#### Influence of sequencing method

Combining microbial community investigation with groundwater chemistry in subsurface environments is essential to better understand our ecosystem because many subsurface biogeochemical processes are mediated by subsurface microorganisms. To compare the bacterial and archaeal community compositions between groundwater groups, we performed both clone libraries construction and Illumina MiSeq sequencing of 16S rRNA genes from groundwater samples in springs or wells (Table S2). In a previous study, soil microbial community analysis using two different sequencing platform (i.e., clone libraries vs. pyrosequencing on a Roche 454 FLX machine) showed some similar results between methods, but also indicated critical differences in microbial community compositions (Jones et al., 2009).

Proteobacteria was the most abundant taxa in all water samples regardless of sequencing platform used, but bacterial communities followed by Proteobacteria were different between the methods (Table S3). In addition, although groundwater samples were extracted under the same conditions and analyzed using Illumina MiSeq platform, microbial community composition was different depending on primer sets used, which was consistent with the previous observation by Tremblay et al. (2015). Before MiSeq run, we performed RDP's probe match for each primer to evaluate the bias associated with each primer pair. Among bacteria, primer 341F (0.95 fraction) covered slightly more than primer 515F (0.93 fraction) (Table S4) and primer 341F generated more bacterial sequences than 515F in all samples (Table S2).

In general, analyses of MiSeq sequencing and clone library data yielded similar results. However, Illumina MiSeq sequencing revealed the greater diversity compared to clone library sequencing (Table S2). In addition, the MiSeq sequencing with 341F-805R yielded greater sequence coverage up to 10-fold depth than that with 515F-805R′. Therefore, we discussed the results of bacterial diversity and taxonomic assignments using the MiSeq sequencing with 341F-805R hereafter.

#### Bacterial diversity and composition

**Figure 2:**
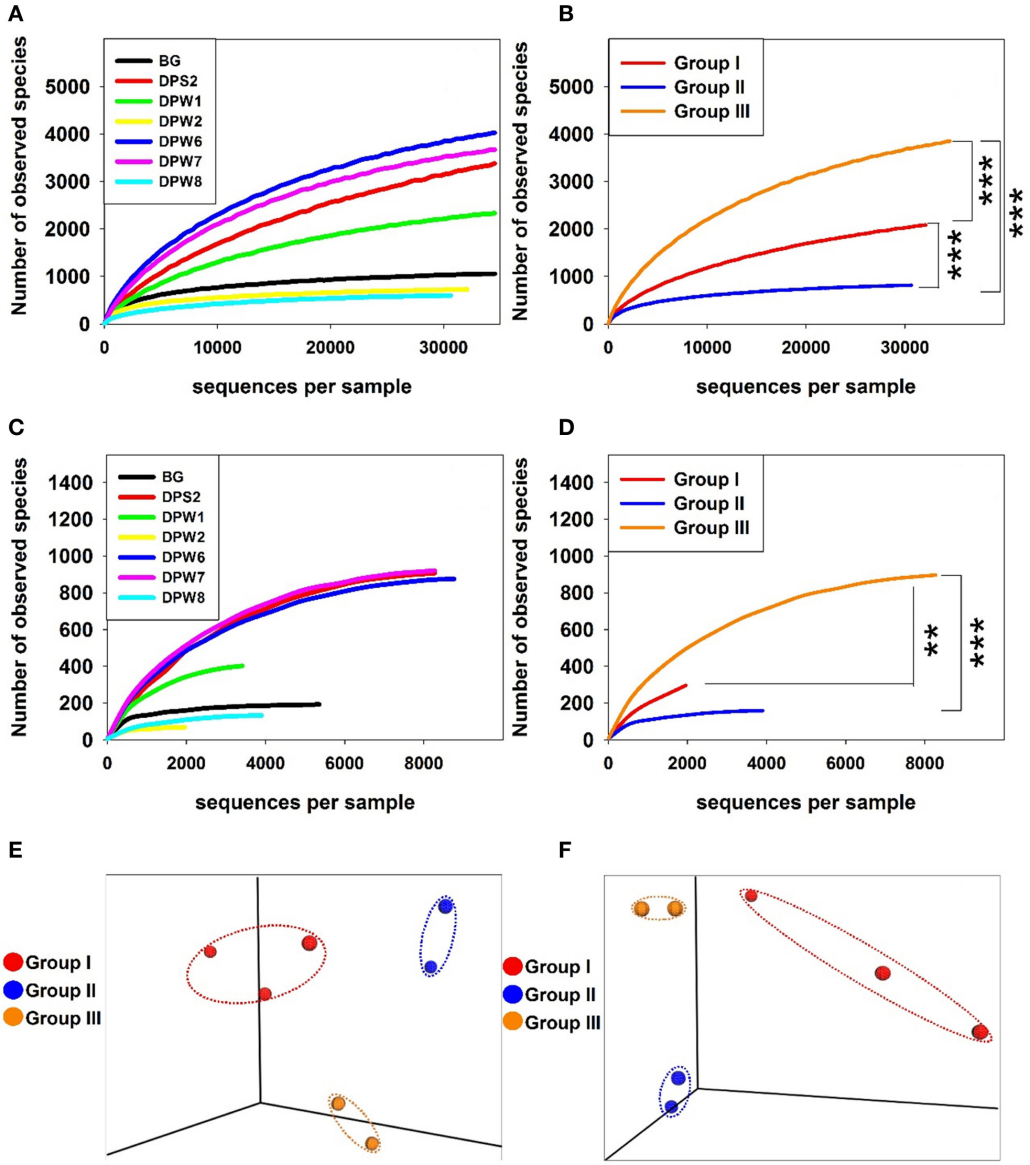
Rarefaction plots of the bacterial **(A,B)** and archaeal **(C,D)** communities in groundwater samples. Three-dimensional principle coordinate analysis (PCoA) plots based on UPGMA (Unweighted Pair Group Method with Arithmetic Mean) data **(E,F)**. Rarefaction curves display the number of operational taxonomic units defined with 97% detected based on the sampling intensity of the libraries. Rarefaction curves of bacteria display the number of observed species obtained from individual samples with increasing sequencing effort **(A)** and compare the average values obtained from the sample included in each group **(B)**. Rarefaction curves of archaea display the number of observed species obtained from individual samples **(C)** and compare the average values obtained from the sample included in each group **(D)**. Mann-Whitney rank sum test were used to identify statistically significant differences between two groups (^***^p < 0.001 and ^**^p < 0.01). PCoA plots were generated from the weighted UniFrac analysis of the bacterial **(E)** archaeal **(F)** sequences.

Analysis of rarefaction curves (plots of the number of species as a function of the number of individuals sampled) indicated the observed species at 97% similarity cut-off. The steep slope in all cases suggested that some fraction of the species diversity remains to be discovered (Figure 2). The rarefaction curves showed that the bacterial diversity was different between groundwater types. A rarefaction curve of groundwater samples influenced by high CO_2_ concentrations revealed lower diversity, suggesting that elevated CO_2_ level can increase the selective pressure for specific bacterial community (Figures 2A,B). Bacterial diversity indices (observed species) significantly increased from group II → group I → group III (Figure 2B). DPW-8, BG, and DPW-2 having high CO_2_ indicated very low number of observed species (<1,100) (Table 2A). Chao1 and Shannon indices calculated based on OTUs also showed relatively low diversity in DPW-8 (620.4 and 5.0, respectively), BG (1160.6 and 7.5), and DPW-2 (790 and 6.5). Meanwhile, DPW-6 and DPW-7 in low-CO_2_ control group III showed a very high number of observed species (>4,000). The Chao1 and Shannon indices of DPW-6 (4,499.6 and 9.5, respectively) and DPW7 (4,096.7 and 8.1) also indicated a relatively high diversity.

**Table 2:** Comparison of bacterial **(A)** and archaeal **(B)** diversity richness and evenness in MiSeq sequencing with 341F-805R.

**Group**	**Name**	**PD_whole_tree**	**Chao1**	**Shannon**	**Observed species**	**Simpson**	**ACE**	**Goods coverage (%)**
**A**								
Group I	DPS2	292.2	4, 737.1	8.3	4,679	0.987	4, 894.0	99.5
	DPW1	241.1	2, 761.4	6.9	2,648	0.941	2, 927.1	99.2
	DPW2	120.3	790.0	6.5	728	0.940	827.8	99.6
Group III	DPW6	458.9	4, 499.6	9.5	4,408	0.992	4, 696.3	99.0
	DPW7	436.2	4, 096.7	8.1	4,011	0.939	4, 257.8	99.1
Group II	DPW8	104.1	620.4	5.0	599	0.868	654.8	99.7
	BG	177.2	1, 160.6	7.5	1,079	0.983	1, 226.6	99.5
**B**								
Group I	DPS2	144.6	911.0	5.9	908	0.878	928.2	99.4
	DPW1	92.4	409.6	6.1	406	0.918	424.9	99.0
	DPW2	31.2	69.7	4.7	69	0.937	72.2	99.7
Group III	DPW6	205.6	879.6	7.1	876	0.960	901.1	99.4
	DPW7	199.0	927.3	7.4	924	0.977	947.2	99.4
Group II	DPW8	41.0	133.4	3.7	133	0.824	136.0	99.8
	BG	72.9	193.9	6.0	193	0.959	198.7	99.8

**Figure 3:**
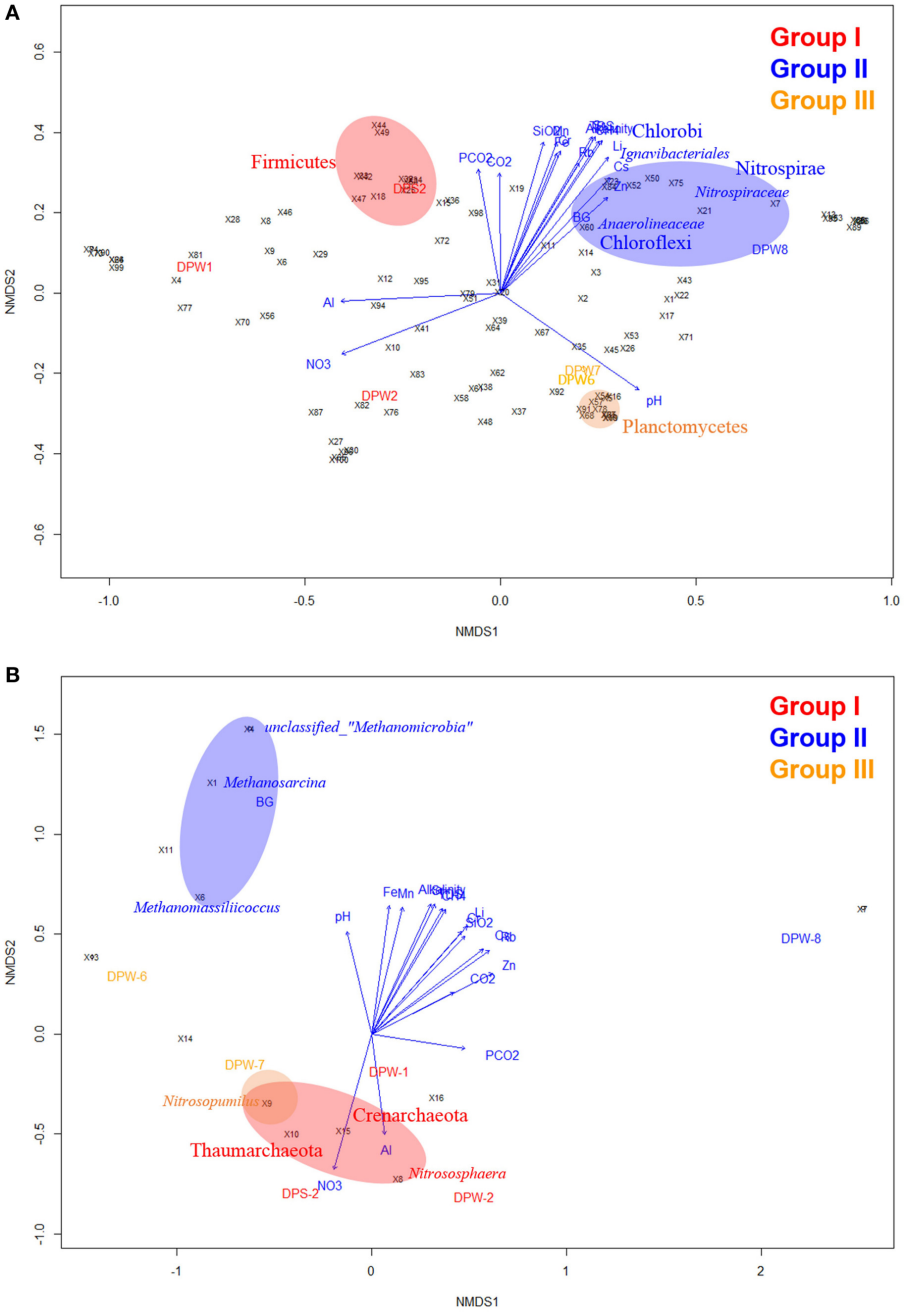
Non-metric multidimensional scaling (NMDS) plot of environmental variables and microbial community compositions in groundwater. NMDS analysis within the vegan package of R software package based on dissimilarities calculated using the Bray–Curtis index of bacterial **(A)** archaeal **(B)** communities composition for the relative abundance of each OTU in relation to the environmental variables. The direction and length of the vectors of groundwater factors from Table 1 are computed by Bray–Curtis distances the “envfit()” function in the vegan package. Each sampling location is coded by the color text (green, group I; red, group II; and blue, group III). The number after the symbol “×” indicates the specific bacterial and archaeal taxa which are shown in Tables S5, S7, respectively.

To confirm the variation of the species composition in this area, beta diversity calculated based on UniFrac analysis was compared between groundwater samples. PCoA plots based on beta diversity displayed three clearly distinguishable groups, which correspond to differences in water quality for bacteria (Figure 2E). In a complimentary analysis, NMDS analysis using top 100 bacterial genera (Table S5) results in the same clustering together (Figure 3A). There were significant differences (*r*^2^ = 0.73, *P* < 0.01) between bacterial communities in groundwater according to their group as determined by PERMANOVA analysis based on Bray-Curtis distance matrix (Anderson, 2001). Environmental variables were overlaid to the plot to confirm whether geochemical factors affect community structure (Figure 3A). Among factors we tested, TDS, alkalinity, Sr, Li, Cs, and CH_4_ showed some significant relationships with community compositions (0.54 < *r*^2^ < 0.70, *P* < 0.1). The microbial community in group II was closely related to TDS, alkalinity, Sr, Li, Cs, and CH_4_, while that in group III was correlated with relatively high pH. In addition, CO_2_ showed close relationships with group I and group II suggesting that CO_2_ may control bacterial community compositions in these groups.

**Figure 4:**
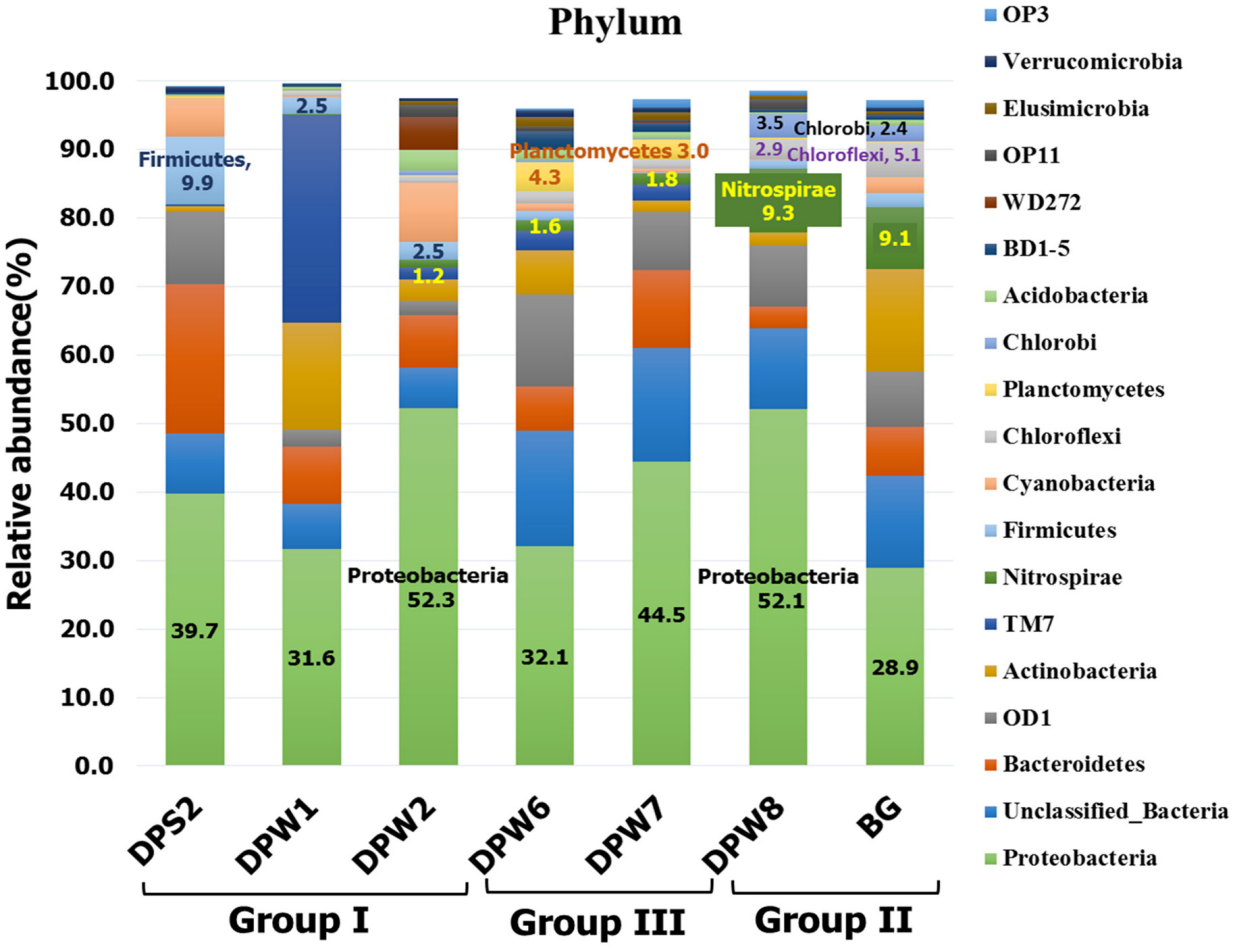
Comparison of the dominant bacterial taxa at the phylum level. The dominant microbes at the phylum level are defined as the taxa had relative abundances of the average value of all groundwater samples >0.5%.

Bacterial analysis by MiSeq sequencing with 341F-805R showed a total of 36 phyla as the following order of average relative abundances; Proteobacteria (40.2%) > Unclassified Bacteria (11.4%) > Bacteriodetes (9.4%) > Candidate division OD1 (7.7%) > Actinobacteria (6.4%) > Candidate division TM7 (5.4%) > *Nitrospirae* (3.3%) > Firmicutes (2.8%) > Cyanobacteria (2.6%) > Chloroflexi (1.8%) (Figure 4 and Table S3C). Proteobacteria was the most abundant phylum in all samples, but ranged broadly between 28.9 and 52.3%. Substantial variation of bacterial community compositions at the phylum level between water samples is not astonishing because different bacterial communities inhabit different environments. However, there are common taxa in specific groundwater group samples. For example, Firmicutes were dominant in group I (5.0 ± 4.3%) and Planctomycetes were abundant in group III (3.7 ± 0.9%) compared to other group samples, while *Nitrospirae* (9.2 ± 0.1%), Chloroflexi (4.0 ± 1.6%), and Chlorobi (3.0 ± 0.8%) were predominant in group II groundwater samples (Figures 3A, 4, Tables S3C, S6A–F). These results suggest that the specific bacterial phyla linked closely to the specific groundwater quality.

The predominance of the phylum *Nitrospirae* in group II was notable (Table S3C). *Nitrospiraceae* is the only established family in this phylum and contains three genera including *Nitrospira* (chemolithoautotrophic aerobic nitrite oxidizer), *Leptospirillum* (chemolithoautotrophic aerobic and acidophilic ferrous iron oxidizers), and *Thermodesulfovibrio* (anaerobic, thermophilic, chemoorganoheterotrophic or hydrogenotrophic sulfate reducers) (Table S6C). Although *Nitrospiraceae* has been isolated from and observed in ocean water, freshwater, aquarium water, marine sediment, soils, wastewater treatment plants, and a corroded iron pipe in a heating system (Watson et al., 1986; Ehrich et al., 1995; Daims et al., 2001; Altmann et al., 2003; Daims, 2014), their predominance in the group II groundwater samples (i.e., not thermophilic temperature, anaerobic, no nitrate) is enigmatic. The order Ignavibacteriales of phylum Chlorobi was also notable in group II (DPW-8; 3.5%, BG; 2.3%, respectively) (Table S6E). The family members in the order Ignavibacteriales are known to play important role in nitrogen cycle (Tian et al., 2015) including ammonification, nitrogen fixation and nitrification, and sulfur-oxidizing autotrophic denitrifying (Zhang et al., 2015).

Relatively high abundance of Chloroflexi, particularly *Anaerolineaceae*, was observed in group II (DPW-8; 2.2%, BG; 3.6%, respectively) (Table S6D). *Anaerolineaceae* has been reported as the most dominant taxa syntrophically associated with methanogenic archaea after long term incubation of methanogenic alkanes-degrader (Liang et al., 2015), and also belonged to dominant community in microbial electrolysis anaerobic digestion reactor system for accelerated methane production (Liu et al., 2016). The presence of *Anaerolineaceae* with methanogenic archaea in group II of our study are also consistent with those studies.

Group I groundwater samples (low pH, low TDS, and high CO_2_) showed relatively high abundance of family *Comamonadaceae* (15, 8.7, and 9.6% in DPS-2, DPW-1, and DPW-2, respectively) (Table S6F). The result is consistent with a field-scale CO_2_ geosequestration study that after CO_2_ injection the relative abundance of *Comamonadaceae* increased with changes in the associated variables (e.g., pH, temperature and salinity) (Mu et al., 2014).

#### Archaeal diversity and composition

For archaeal community analysis, we also compared the sequencing results from three different methods (i.e., clone library with 109F-958R and MiSeq with 515F-805R′ or 341F-805R). Like bacterial analysis, we assessed the compatibility of two different primer sets for detecting archaeal sequences. The result of RDP's probe match showed that 515F covered 0.56 fraction of archaeal sequences but 341F did not cover at all (Table S4). However, 341F-805R indeed revealed a better depth of sequences than 515F-805R′ in this study (Table S2B). Table 2B summarizes the number of observed species and various diversity indices of archaea. Although the depth of sequences was reasonable for determining archaeal diversity, the sequencing result even with 341F-805R did not show the accurate information of archaeal community compositions because the most reads were unassigned archaeal OTUs in both reference databases, RDP and SILVA. The taxonomic assignment was better in clone library analysis than in sequencing either with 515F-805R′ or 341F-805R primer sets. Therefore, we discussed hereafter archaeal diversity based on the sequencing results with 341F-805R, but archaeal taxonomy using the clone library sequencing results with 109F-958R.

Similar to bacterial diversity, the alpha diversity slopes of archaea in the presence of high CO_2_ were significantly lower than those in low CO_2_ water. Archaeal diversity indices (observed species) also significantly increased from group II → group I → group III. The species diversity in partial pressure with no or relatively low concentrations of CO_2_ was similar each other, but the presence of high CO_2_ (>3,200 mg L^−1^) decreased the number of observed species (Figures 2C,D).

The species richness of DPW-8 and BG in group II and DPW-2 in group I was low, which might be correlated with high CO_2_ concentrations. DPW8 and BG showed low diversity indices of observed species, Chao1, and ACE (Table 2B). Unlike bacterial results, DPW-2 showed the lowest values of observed species (69), Chao1 (69.7), and ACE (72.2).

The beta diversity analysis for better understanding variation of species composition in archaeal community showed the three separated clusters in 3D-PCoA plot (Figure 2F). Similar to the bacterial community structure, DPW-8 and BG in group II and DPW-6 and DPW-7 in group III were very closely distanced, but DPS-2, DPW-1, and DPW-2 in group I were dispersed. This result suggests that microbial diversity in springs and wells is strongly affected by the level of CO_2_ concentrations.

Archaeal community analysis by clone library construction with 109F-958R showed a total of 6 phyla. The result of NMDS from archaeal data (Figure 3B) revealed that relative abundance of top 16 archaeal genera (Table S7) across samples was linked distinctly to each groundwater group (*r*^2^ = 0.53, *P* = 0.11). In addition, archaeal community compositions showed significant correlation with several environmental variables including alkalinity, Li, Rb, and Cs (*r*^2^ > 0.89, *P* < 0.01) and TDS, SiO_2_, ${\text{NO}}_{3}^{-}$, Sr, Cr, and CH_4_ (*r*^2^ > 0.82, *P* < 0.05).

**Figure 5:**
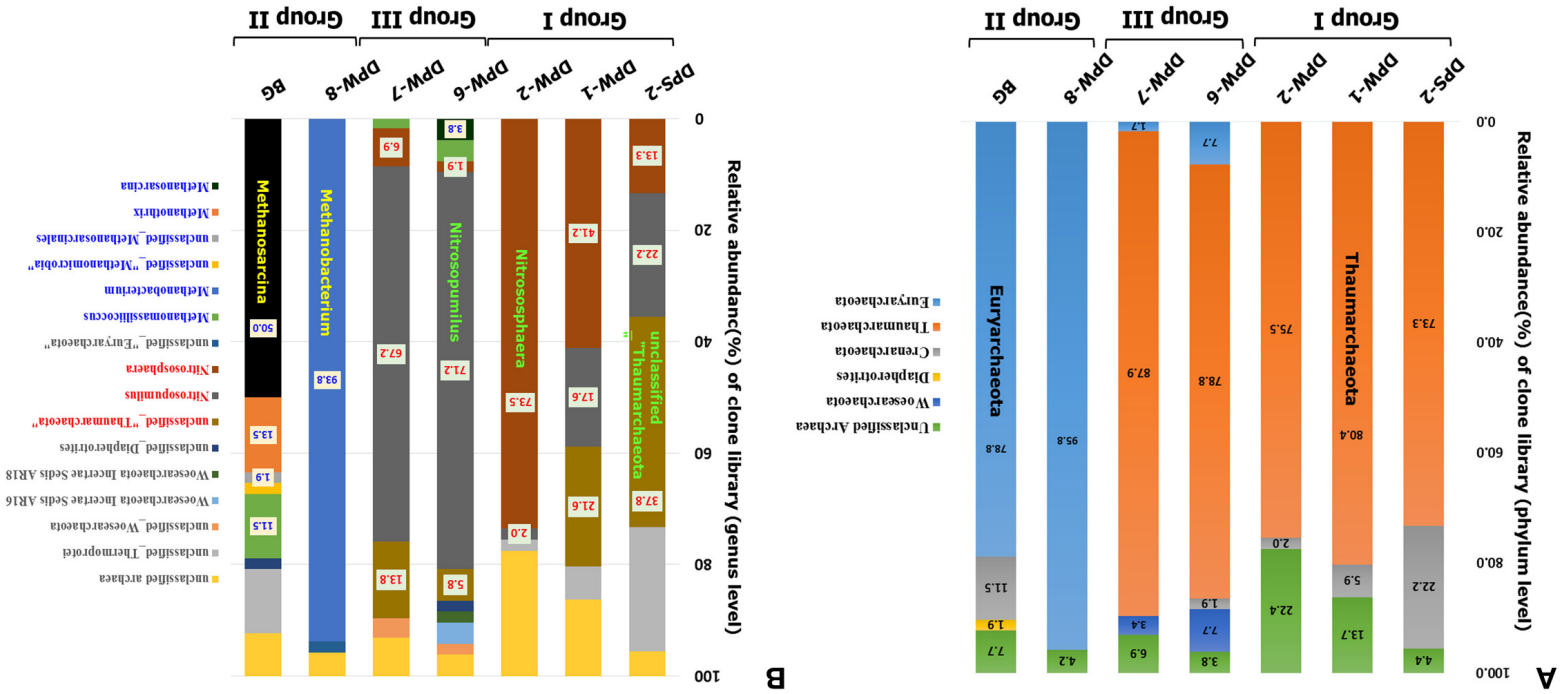
Comparison of the dominant archaeal taxa at the phylum **(A)** and genus **(B)** level. All microbes at the phylum **(A)** and genus **(B)** level are represented, including ammonia oxidizing archaea (red) and methane producing or methane oxidizing archaea (blue).

Taxonomic distribution of archaeal community was distinct in each group (Figure 5 and Table S8). Euryarchaeota were dominated in group II groundwater samples, while Thaumarchaeota containing ammonia-oxidizing archaea (AOA) were predominant (73–88%) in group I and III groundwater samples. Ammonia-oxidizing bacteria (AOB) (Jiang and Bakken, 1999) and AOA (Könneke et al., 2005) are known to be able to oxidize NH_3_ to ${\text{NO}}_{2}^{-}$. High abundance of AOA (e.g., *Nitrososphaera* and *Nitrosopumilus*) suggests that nitrification occurs to some extent in these environments. *Nitrososphaera* as acidophilic or thermophilic AOA usually inhabit terrestrial environments (i.e., soil), whereas *Nitrosopumilus* as mesophilic AOA usually presented in aquatic environments (i.e., marine water, freshwater and wastewater) (Spang et al., 2010; Pester et al., 2012). In addition, *Nitrobacter*, a genus of well-known nitrite oxidizing bacteria (NOB) (i.e., ${\text{NO}}_{2}^{-}$ → ${\text{NO}}_{3}^{-}$), was observed in group I and III (i.e., DPS-2:0.1%, DPW-1:0.8%, DPW-6:0.1% and DPW-7:0.1%) (Teske et al., 1994). The presence of NOB as well as AOA with substantial amount of nitrate only in group I and III indicate the nitrification (ammonia and nitrite oxidation) in this environment (i.e., NH_3_ → ${\text{NO}}_{2}^{-}$ → ${\text{NO}}_{3}^{-}$).

Groundwater samples with high CO_2_ and CH_4_ showed clear differences in archaeal community compositions at the phylum level (Figure 5A). High abundance of methanogens in archaeal community in DPW-8 and BG coupled with presence of CH_4_ suggests that methanogenesis is actively ongoing in group II groundwaters. Relative abundance of Euryarchaeota related to methanogenesis in DPW-8 and BG is 95.8 and 78.8%, respectively (Figure 5A), which corresponds well to the presence of CH_4_ in these samples (35 and 22 mg L^−1^, respectively) (Table 1). Interestingly, genus level compositions of archaeal community in between DPW-8 and BG were very different. Most archaeal sequences in samples from DPW-8 grouped in *Methanobacterium* (93.8%) whereas in samples for BG, archaeal sequences grouped in *Methanosarcina* (50%), *Methanothrix* (13.5%), and *Methanomassiliicoccus* (11.5%).

There are three known metabolic pathways for methanogenesis (Whitman et al., 2006); (1) hydrogenotrophic (e.g., 4H_2_ + CO_2_ → 2H_2_O + CH_4_), (2) acetotrophic (e.g., CH_3_COOH → CO_2_ + CH_4_), and (3) methylotrophic (e.g., 4CH_3_OH → 3CH_4_ + CO_2_ + 2H_2_O). *Methanobacterium* species are strict anaerobes and hydrogenotrophic methanogens capable of using H_2_ (Schirmack et al., 2014). Meanwhile, *Methanosarcina* and *Methanosaeta* (“*Methanothrix*”) are two genera among methanogens capable of using acetate to produce methane via acetoclastic methanogenesis (Zinder et al., 1984; Jetten et al., 1992; Kendall and Boone, 2006). Therefore, differences in the methanogenic archaeal community between DPW-8 and BG suggests that the methane in samples from each site may have been generated by different methanogenic pathways. Given that groundwater samples from each site had similar major ion chemistry and CO_2_ content, we hypothesize that differences in methanogenic communities may be related to differences in DOC concentration in BG (4.7 mg L^−1^) compared to that in DPW-8 (1.7 mg L^−1^).

### CO_2_ as a control on microbial community composition

Our findings indicate that the diverse geochemical conditions at the field sites influence the microbial community in different ways. Based on the taxonomy of sequences in our samples, the function of the microbial community appears to be more closely tied to the redox state of the environment rather than CO_2_ concentration. For example, we find species that participate in nitrogen cycling in samples from group I and III that contained small amounts of DO and significant nitrate concentrations but little ferrous iron and methane. In contrast, we see high relative abundances of methanogens and metal reducers in samples from group II, which contained lower oxygen concentrations and undetectable nitrate levels. Consistent with this interpretation, our beta diversity calculations indicate a significant relationship between the composition of the microbial community in our samples and CH_4_ concentration.

In contrast, the alpha diversity of our samples is more closely linked to CO_2_ concentration than redox state. For both archaea and bacteria, samples from the low-CO_2_ site (group III) had the most diverse communities. A similar relationship has been observed in soils. Three independent studies have shown that microbial community diversity in soil is closely tied to pH on a global scale (Fierer and Jackson, 2006; Jones et al., 2009; Lauber et al., 2009). In aqueous systems, pH is closely linked to CO_2_ concentration. Thus, to some extent, the significant relationship we observed between CO_2_ concentration and alpha diversity may simply reflect variation in pH. In addition, salinity may have also influenced alpha diversity. Among samples from the high-CO_2_ groups, those from group I had the lowest diversity and the highest salinity, despite having a less extreme pH than samples from group II.

### Environmental significance

#### Carbon budget

Our findings imply that microbial reactions in the shallow aquifer are influencing the carbon budget of the system in a variety of ways. Reaction of CO_2_ with carbonate and silicate minerals, as discussed previously, generates carbonate alkalinity as well as divalent metals (e.g., Ca^2+^, Mg^2+^, Fe^2+^, and etc.) that can ultimately precipitate as carbonate minerals (e.g., calcite, dolomite, siderite, and etc.). Where those reactions occur, CO_2_ would be trapped in mineral form, rather than emitted to the atmosphere. The microbial community may enhance mineral trapping through microbial generation of carbonate alkalinity. Many microbial reactions consume protons. As such, they drive CO_2_ hydrolysis forward and thus convert CO_2_ into bicarbonate. Microbial iron reduction, which may be generating the ferrous iron detected in samples from group II, is particularly effective at generating alkalinity because the reaction consumes several protons (Kirk et al., 2013). Microorganisms may also enhance carbon trapping by providing surfaces serve as nucleation sites for carbonate mineralization (Mitchell et al., 2010; Benzerara et al., 2011).

In addition to converting CO_2_ into carbonate alkalinity or carbonate minerals, microorganisms can also transform the carbon in CO_2_ into organic forms. Although the Wood-Ljungahl pathway isn't widespread within the Chloroflexi, two Chloroflexi species have been shown to use the Wood–Ljungdahl pathway for CO_2_ fixation and play an important role in sediment carbon cycling (Hug et al., 2013). Therefore, the Chloroflexi may be helping to trap CO_2_ as organic carbon in the subsurface at the site. In contrast, reduction of CO_2_ to methane by hydrogenotrophic methanogens could result in loss of carbon as methane from the shallow subsurface. A relatively high abundance of Euryarchaeota associated with methanogenesis was observed in group II samples that also contained significant levels of CH_4_. Methane is a potent greenhouse gas and its global warming potential is considered 25 times higher than that of CO_2_ (Oppermann et al., 2010). Thus understanding microbial controls and developing engineered controls on its release from deep subsurface environments to the atmosphere is an important research need.

#### Implication for biological monitoring of CO_2_ leakage

Alterations in microbial community structure have been observed during CO_2_ storage in rhizosphere (Grayston et al., 1998) and soil (Rillig et al., 1997; Mayr et al., 1999; Horz et al., 2004) and thus can be used as a sign of CO_2_ leakage. The previous studies of field-scale CO_2_ geosequestration showed that the relative abundance of *Comamonadaceae* increased early after CO_2_ injection with decreases in pH and mobilization in minor elements, such as Fe and Mn, temperature and salinity) (Mu et al., 2014; O'Mullan et al., 2015). Our study also showed clear relationships between microbial community compositions and CO_2_-rich waters with relatively short-term or long-term water-rock interactions after the influx of CO_2_ gases. As shown in group I case, the detection of *Comamonadaceae* in groundwater samples collected from near CO_2_ storage sites would be an indication of the location influenced by CO_2_ leakage with short-term water-rock reaction. The observation of methanogens, *Nitrospirae*, and *Anaerolineaceae* as shown in group II case may be an indication of the sites undergone both more CO_2_ leakage and long-term water-rock reaction and biogeochemical reaction. Also, the decrease in bacterial and archaeal diversity in groundwater with increase in CO_2_ would be an indication that CO_2_ release from the storage sites is in progress and impacts groundwater microbial community. However, the differences in bacterial and archaeal diversity and compositions would be site specific. Therefore, long-term and periodical monitoring of microbial community in subsurface environments are necessary.

## Conclusions

This study investigated microbial community diversity and compositions in CO_2_-rich groundwater samples to understand the impact of CO_2_ released from underground storage sites on subsurface biogeochemical processes. The water quality and groundwater microbial community data collected from natural analog sites similar to the sites impacted by high to low level of CO_2_ leakage suggest that the function of the microbial community appears to be more closely tied to the redox state of the environment rather than CO_2_ concentration. On the other hand, the bacterial and archaeal diversities were remarkably lower with high concentrations of CO_2_ and low pH. Specific microbial groups, *Comamonadaceae*, predominated in CO_2_-rich and low pH environments, whereas methanogens, uncultured *Nitrospiraceae*, and *Anaerolineaceae* were abundant in CH_4_-/dissolved iron-rich and nitrate-poor groundwater environments. The data suggest that the field sites for this study were a good natural laboratory to explore the relationships between microbial communities and geochemistry. Assessment of microbial community compositions in groundwater can be an effective tool for monitoring CO_2_ leakage from underground storage reservoirs and provides insight into biogeochemical changes that occurred during CO_2_ storage. Further investigation in the field sites of this study will expand our understanding of short- or long-term CO_2_ leakage impact on soil and groundwater ecosystem.

## Author contributions

B-YC, G-TC, and MJK designed the studies. BH, B-YC, G-TC, and MJK performed the experiments. BH, B-YC, G-TC, MFK, and MJK analyzed the data. BH, B-YC, MFK, and MJK wrote the manuscript.

### Conflict of interest statement

The authors declare that the research was conducted in the absence of any commercial or financial relationships that could be construed as a potential conflict of interest.
