# Supplementary material for: Geochemical Influence on Microbial Communities at CO2-Leakage Analog Sites
Source: Front Microbiol. 2017 Nov 9;8:2203. doi: 10.3389/fmicb.2017.02203 (PMC5684959; doi:10.3389/fmicb.2017.02203)
Supplement: Supplementary file 1 [file Table1.DOCX]

S1 Table. Sequencing statistics according to two different taxonomic reference databases.

| **MiSeq (341F-805R)** | | | | |
| --- | --- | --- | --- | --- |
|  | **Bacteria** | | **Archaea** | |
| **Ref. database** | **RDP** | **SILVA** | **RDP** | **SILVA** |
| **No. samples** | 7 | 7 | 7 | 7 |
| **No. observations** | 13880 | 13958 | 122 | 3101 |
| **Total count** | 355672 | 356604 | 1938 | 41169 |
| **Counts/sample summary** | | | | |
| **Min** | 30681 | 30714 | 22 | 1974 |
| **Max** | 96054 | 96077 | 545 | 8832 |
| **Median** | 51612 | 51737 | 223 | 5409 |
| **Mean** | 50810 | 50943 | 277 | 5881 |
| **Std. dev.** | 20620 | 20598 | 223 | 2581 |
| **Counts/sample detail** | | | | |
| **DPS2** | 96054 | 96077 | 27 | 8465 |
| **DPW1** | 55254 | 55262 | 22 | 3694 |
| **DPW2** | 32484 | 32518 | 94 | 1974 |
| **DPW6** | 51698 | 52141 | 501 | 8832 |
| **DPW7** | 51612 | 51737 | 223 | 8681 |
| **DPW8** | 30681 | 30714 | 526 | 4114 |
| **BG** | 37889 | 38155 | 545 | 5409 |
