# Supplementary material for: Geochemical Influence on Microbial Communities at CO2-Leakage Analog Sites
Source: Front Microbiol. 2017 Nov 9;8:2203. doi: 10.3389/fmicb.2017.02203 (PMC5684959; doi:10.3389/fmicb.2017.02203)
Supplement: Supplementary file 2 [file Table2.DOCX]

S2 Table. Sequencing statistics of three different sequencing analysis

1. Bacteria

| **Bacteria** | **Clone library** | **MiSeq (**515F-806R**)** | **MiSeq (**341F-805R**)** |
| --- | --- | --- | --- |
| **No. samples** | 7 | 7 | 7 |
| **Total count** | 401 | 9096 | 356604 |
| **Counts/sample summary** | | | |
| **Min** | 50 | 656 | 30714 |
| **Max** | 63 | 2555 | 96077 |
| **Median** | 57 | 1101 | 51737 |
| **Mean** | 57 | 1299 | 50943 |
| **Std. dev.** | 4 | 624 | 20598 |
| **Counts/sample detail** | | | |
| **DPS2** | 56 | 2555 | 96077 |
| **DPW1** | 63 | 1743 | 55262 |
| **DPW2** | 56 | 698 | 32518 |
| **DPW6** | 59 | 1399 | 52141 |
| **DPW7** | 60 | 944 | 51737 |
| **DPW8** | 57 | 1101 | 30714 |
| **BG** | 50 | 656 | 38155 |

1. Archaea

| **Archaea** | **Clone library** | **MiSeq (**515F-806R**)** | **MiSeq (**341F-805R**)** |
| --- | --- | --- | --- |
| **No. samples** | 7 | 7 | 7 |
| **Total count** | 355 | 996 | 41169 |
| **Counts/sample summary** | | | |
| **Min** | 45 | 45 | 1974 |
| **Max** | 58 | 225 | 8832 |
| **Median** | 51 | 124 | 5409 |
| **Mean** | 51 | 142 | 5881 |
| **Std. dev.** | 4 | 65 | 2581 |
| **Counts/sample detail** | | | |
| **DPS2** | 45 | 220 | 8465 |
| **DPW1** | 51 | 118 | 3694 |
| **DPW2** | 49 | 45 | 1974 |
| **DPW6** | 52 | 189 | 8832 |
| **DPW7** | 58 | 124 | 8681 |
| **DPW8** | 48 | 225 | 4114 |
| **BG** | 52 | 75 | 5409 |
