# Supplementary material for: Geochemical Influence on Microbial Communities at CO2-Leakage Analog Sites
Source: Front Microbiol. 2017 Nov 9;8:2203. doi: 10.3389/fmicb.2017.02203 (PMC5684959; doi:10.3389/fmicb.2017.02203)
Supplement: Supplementary file 3 [file Table3.DOCX]

S3 Table. Bacterial community compositions of clone library and MiSeq sequencing (phylum level)

1. Clone library

| **Group** | **I** | | | | **III** | | | **II** | | | **All_average** |
| --- | --- | --- | --- | --- | --- | --- | --- | --- | --- | --- | --- |
| **Sample Name Phylum** | **DPS2** | **DPW1** | **DPW2** | **Average** | **DPW6** | **DPW7** | **Average** | **DPW8** | **BG** | **Average** |  |
| **Proteobacteria** | 80.4 | 54 | 64.3 | 66.2 | 69.5 | 65 | 67.3 | 84.2 | 56 | 70.1 | 67.6 |
| **Unclassified Bacteria** | 0 | 0 | 1.8 | 0.6 | 8.5 | 20 | 14.3 | 7 | 24 | 15.5 | 8.8 |
| **Actinobacteria** | 0 | 15.9 | 0 | 5.3 | 0 | 0 | 0.0 | 0 | 6 | 3.0 | 3.1 |
| **Cyanobacteria/Chloroplast** | 0 | 0 | 17.9 | 6.0 | 0 | 0 | 0.0 | 0 | 4 | 2.0 | 3.1 |
| **Bacteroidetes** | 14.3 | 9.5 | 0 | 7.9 | 3.4 | 3.3 | 3.4 | 0 | 6 | 3.0 | 5.2 |
| **Candidatus Saccharibacteria** | 0 | 12.7 | 1.8 | 4.8 | 0 | 0 | 0.0 | 0 | 0 | 0.0 | 2.1 |
| **Nitrospirae** | 0 | 1.6 | 7.1 | 2.9 | 8.5 | 3.3 | 5.9 | 0 | 0 | 0.0 | 2.9 |
| **Candidate division WPS-2** | 0 | 0 | 3.6 | 1.2 | 0 | 0 | 0.0 | 0 | 0 | 0.0 | 0.5 |
| **Elusimicrobia** | 0 | 0 | 0 | 0.0 | 0 | 0 | 0.0 | 3.5 | 0 | 1.8 | 0.5 |
| **Firmicutes** | 5.4 | 4.8 | 0 | 3.4 | 1.7 | 0 | 0.9 | 0 | 2 | 1.0 | 2.0 |
| **Acidobacteria** | 0 | 1.6 | 1.8 | 1.1 | 6.8 | 0 | 3.4 | 0 | 0 | 0.0 | 1.5 |
| **Microgenomates(OP11)** | 0 | 0 | 0 | 0.0 | 0 | 3.3 | 1.7 | 1.8 | 0 | 0.9 | 0.7 |
| **Planctomycetes** | 0 | 0 | 0 | 0.0 | 1.7 | 3.3 | 2.5 | 0 | 0 | 0.0 | 0.7 |
| **Latescibacteria(WS3)** | 0 | 0 | 0 | 0.0 | 0 | 0 | 0.0 | 0 | 2 | 1.0 | 0.3 |
| **Parcubacteria(OD1)** | 0 | 0 | 0 | 0.0 | 0 | 0 | 0.0 | 1.8 | 0 | 0.9 | 0.3 |
| **Verrucomicrobia** | 0 | 0 | 0 | 0.0 | 0 | 0 | 0.0 | 1.8 | 0 | 0.9 | 0.3 |
| **Armatimonadetes** | 0 | 0 | 1.8 | 0.6 | 0 | 1.7 | 0.9 | 0 | 0 | 0.0 | 0.5 |

1. MiSeq sequencing using 515F-806R primer set

| **Group** | **I** | | | | **III** | | | **II** | | | **All_average** |
| --- | --- | --- | --- | --- | --- | --- | --- | --- | --- | --- | --- |
| **Sample Name**  **Phylum** | **DPS2** | **DPW1** | **DPW2** | **Average** | **DPW6** | **DPW7** | **Average** | **DPW8** | **BG** | **Average** |  |
| **Proteobacteria** | 33 | 28.8 | 29.9 | 30.6 | 29.5 | 35.2 | 32.4 | 30.6 | 21.8 | 26.2 | 29.8 |
| **Bacteroidetes** | 35.7 | 15.3 | 8.3 | 19.8 | 11.9 | 24.4 | 18.2 | 7.8 | 10.7 | 9.3 | 16.3 |
| **Verrucomicrobia** | 1.9 | 2.9 | 38.3 | 14.4 | 4.1 | 6.8 | 5.5 | 20.5 | 35.1 | 27.8 | 15.7 |
| **Unclassified Bacteria** | 8.6 | 6.8 | 6.3 | 7.2 | 13.2 | 13.1 | 13.2 | 18.2 | 11 | 14.6 | 11.0 |
| **Candidate division TM7** | 0.5 | 35.6 | 0.9 | 12.3 | 5.9 | 5.4 | 5.7 | 0 | 0.2 | 0.1 | 6.9 |
| **Candidate division OD1** | 4.7 | 1 | 0.1 | 1.9 | 7.6 | 4.2 | 5.9 | 2.5 | 2.7 | 2.6 | 3.3 |
| **Actinobacteria** | 0.4 | 4.1 | 4.2 | 2.9 | 2.6 | 1 | 1.8 | 2 | 4.6 | 3.3 | 2.7 |
| **Firmicutes** | 6.3 | 2.5 | 3 | 3.9 | 2.6 | 0.5 | 1.6 | 0.9 | 1.7 | 1.3 | 2.5 |
| **Cyanobacteria** | 6.3 | 0.4 | 4.3 | 3.7 | 1.1 | 0.3 | 0.7 | 1.2 | 3.4 | 2.3 | 2.4 |
| **Nitrospirae** | 0 | 0.1 | 0 | 0.0 | 1.6 | 0.6 | 1.1 | 5.3 | 4 | 4.7 | 1.7 |
| **BD1-5** | 0.6 | 0.5 | 0.6 | 0.6 | 4.8 | 2.6 | 3.7 | 0.7 | 0.2 | 0.5 | 1.4 |
| **Planctomycetes** | 0.5 | 0 | 2.7 | 1.1 | 3.1 | 0.7 | 1.9 | 0.6 | 0.9 | 0.8 | 1.2 |
| **Chlorobi** | 0 | 0 | 0 | 0.0 | 0.2 | 0 | 0.1 | 4.8 | 0.9 | 2.9 | 0.8 |
| **Acidobacteria** | 0.4 | 0.5 | 0.7 | 0.5 | 1.9 | 0.6 | 1.3 | 0.3 | 0.5 | 0.4 | 0.7 |
| **Chloroflexi** | 0 | 0.6 | 0.1 | 0.2 | 1.1 | 0.4 | 0.8 | 0.6 | 0.8 | 0.7 | 0.5 |
| **SM2F11** | 0.5 | 0.2 | 0 | 0.2 | 2.7 | 0.2 | 1.5 | 0 | 0 | 0.0 | 0.5 |
| **Candidate division OP11** | 0 | 0.6 | 0.4 | 0.3 | 0 | 0.7 | 0.4 | 1.5 | 0 | 0.8 | 0.5 |
| **Elusimicrobia** | 0 | 0 | 0 | 0.0 | 1.9 | 0.4 | 1.2 | 0.8 | 0 | 0.4 | 0.4 |
| **Candidate division WS6** | 0 | 0 | 0 | 0.0 | 0.8 | 1.5 | 1.2 | 0 | 0 | 0.0 | 0.3 |
| **Chlamydiae** | 0.2 | 0 | 0 | 0.1 | 1.8 | 0 | 0.9 | 0 | 0.2 | 0.1 | 0.3 |
| **Candidate division OP3** | 0 | 0 | 0 | 0.0 | 0.1 | 0.6 | 0.4 | 0.5 | 0.5 | 0.5 | 0.2 |
| **WCHB1-60** | 0 | 0 | 0 | 0.0 | 0.6 | 0.2 | 0.4 | 0.6 | 0 | 0.3 | 0.2 |
| **TA06** | 0 | 0.1 | 0 | 0.0 | 0 | 0 | 0.0 | 0 | 0.5 | 0.3 | 0.1 |
| **Spirochaetae** | 0 | 0 | 0 | 0.0 | 0.1 | 0.1 | 0.1 | 0.4 | 0 | 0.2 | 0.1 |
| **Lentisphaerae** | 0 | 0 | 0 | 0.0 | 0 | 0 | 0.0 | 0.2 | 0.3 | 0.3 | 0.1 |
| **Gemmatimonadetes** | 0 | 0 | 0 | 0.0 | 0.4 | 0 | 0.2 | 0 | 0 | 0.0 | 0.1 |
| **Candidate division SR1** | 0.4 | 0 | 0 | 0.1 | 0 | 0 | 0.0 | 0 | 0 | 0.0 | 0.1 |
| **Armatimonadetes** | 0 | 0 | 0 | 0.0 | 0 | 0 | 0.0 | 0 | 0.3 | 0.2 | 0.0 |
| **Tenericutes** | 0 | 0 | 0.1 | 0.0 | 0 | 0 | 0.0 | 0 | 0.2 | 0.1 | 0.0 |
| **NPL-UPA2** | 0 | 0 | 0 | 0.0 | 0 | 0.2 | 0.1 | 0 | 0 | 0.0 | 0.0 |
| **SHA-109** | 0.1 | 0 | 0 | 0.0 | 0 | 0 | 0.0 | 0 | 0 | 0.0 | 0.0 |

1. MiSeq sequencing using 341F-805R primer set

| **Group** | **I** | | | | **III** | | | **II** | | | **All_average** |
| --- | --- | --- | --- | --- | --- | --- | --- | --- | --- | --- | --- |
| **Sample Name**  **Phylum** | **DPS2** | **DPW1** | **DPW2** | **Average** | **DPW6** | **DPW7** | **Average** | **DPW8** | **BG** | **Average** |  |
| **Proteobacteria** | 39.7 | 31.6 | 52.3 | 41.2 | 32.1 | 44.5 | 38.3 | 52.1 | 28.9 | 40.5 | 40.2 |
| **Unclassified_Bacteria** | 8.8 | 6.7 | 5.9 | 7.1 | 16.8 | 16.6 | 16.7 | 11.8 | 13.5 | 12.7 | 11.4 |
| **Bacteroidetes** | 21.8 | 8.4 | 7.6 | 12.6 | 6.5 | 11.3 | 8.9 | 3.2 | 7.1 | 5.2 | 9.4 |
| **OD1** | 10.6 | 2.4 | 2.1 | 5.0 | 13.5 | 8.5 | 11.0 | 9 | 8.2 | 8.6 | 7.8 |
| **Actinobacteria** | 0.8 | 15.7 | 3.2 | 6.6 | 6.5 | 1.6 | 4.1 | 1.8 | 14.9 | 8.4 | 6.4 |
| **TM7** | 0.3 | 30.2 | 1.8 | 10.8 | 2.8 | 2.3 | 2.6 | 0.1 | 0 | 0.1 | 5.4 |
| **Nitrospirae** | 0 | 0.2 | 1.2 | 0.5 | 1.6 | 1.8 | 1.7 | 9.3 | 9.1 | 9.2 | 3.3 |
| **Firmicutes** | 9.9 | 2.5 | 2.5 | 5.0 | 1.4 | 0.1 | 0.8 | 1.2 | 2.1 | 1.7 | 2.8 |
| **Cyanobacteria** | 5.6 | 0.1 | 8.7 | 4.8 | 1.1 | 0.5 | 0.8 | 0.2 | 2.2 | 1.2 | 2.6 |
| **Chloroflexi** | 0 | 0.7 | 1 | 0.6 | 1.8 | 1.4 | 1.6 | 2.9 | 5.1 | 4.0 | 1.8 |
| **Planctomycetes** | 0.3 | 0.2 | 0 | 0.2 | 4.3 | 3 | 3.7 | 0.2 | 0.2 | 0.2 | 1.2 |
| **Chlorobi** | 0 | 0.1 | 0.8 | 0.3 | 0.3 | 0.1 | 0.2 | 3.5 | 2.4 | 3.0 | 1.0 |
| **Acidobacteria** | 0.2 | 0.4 | 2.9 | 1.2 | 1.5 | 0.9 | 1.2 | 0.2 | 0.7 | 0.5 | 1.0 |
| **BD1-5** | 0.3 | 0.1 | 0 | 0.1 | 2.7 | 1.4 | 2.1 | 0.5 | 0.7 | 0.6 | 0.8 |
| **WD272** | 0 | 0 | 4.8 | 1.6 | 0 | 0.1 | 0.1 | 0 | 0.1 | 0.1 | 0.7 |
| **OP11** | 0 | 0.2 | 1.8 | 0.7 | 0.6 | 0.4 | 0.5 | 1.4 | 0.1 | 0.8 | 0.6 |
| **Elusimicrobia** | 0 | 0 | 0.5 | 0.2 | 1.4 | 1 | 1.2 | 0.5 | 0.4 | 0.5 | 0.5 |
| **Verrucomicrobia** | 0.8 | 0.2 | 0.5 | 0.5 | 0.9 | 0.7 | 0.8 | 0 | 0.5 | 0.3 | 0.5 |
| **OP3** | 0 | 0 | 0 | 0.0 | 0.4 | 1.2 | 0.8 | 0.7 | 1.1 | 0.9 | 0.5 |
| **Chlamydiae** | 0.3 | 0 | 0 | 0.1 | 1.6 | 0.4 | 1.0 | 0 | 0 | 0.0 | 0.3 |
| **Armatimonadetes** | 0.1 | 0 | 1 | 0.4 | 0.1 | 0.1 | 0.1 | 0.1 | 0.6 | 0.4 | 0.3 |
| **SM2F11** | 0.2 | 0.1 | 0.6 | 0.3 | 0.6 | 0.3 | 0.5 | 0 | 0 | 0.0 | 0.3 |
| **Spirochaetae** | 0 | 0 | 0.3 | 0.1 | 0.1 | 0.1 | 0.1 | 0.6 | 0.2 | 0.4 | 0.2 |
| **WCHB1-60** | 0 | 0.1 | 0.1 | 0.1 | 0.4 | 0.1 | 0.3 | 0.4 | 0 | 0.2 | 0.2 |
| **TA06** | 0 | 0 | 0 | 0.0 | 0.1 | 0 | 0.1 | 0 | 0.9 | 0.5 | 0.1 |
| **WS6** | 0 | 0 | 0 | 0.0 | 0.1 | 0.8 | 0.5 | 0 | 0 | 0.0 | 0.1 |
| **NPL-UPA2** | 0 | 0 | 0 | 0.0 | 0.2 | 0.5 | 0.4 | 0 | 0 | 0.0 | 0.1 |
| **WS3** | 0 | 0 | 0 | 0.0 | 0.1 | 0.1 | 0.1 | 0 | 0.3 | 0.2 | 0.1 |
| **Lentisphaerae** | 0 | 0 | 0 | 0.0 | 0 | 0 | 0.0 | 0.1 | 0.3 | 0.2 | 0.1 |
| **Gemmatimonadetes** | 0 | 0 | 0 | 0.0 | 0.3 | 0 | 0.2 | 0 | 0 | 0.0 | 0.0 |
| **BRC1** | 0 | 0 | 0 | 0.0 | 0.1 | 0 | 0.1 | 0.1 | 0.1 | 0.1 | 0.0 |
| **Fibrobacteres** | 0 | 0 | 0 | 0.0 | 0 | 0 | 0.0 | 0 | 0.1 | 0.1 | 0.0 |
| **SR1** | 0.2 | 0 | 0 | 0.1 | 0 | 0 | 0.0 | 0 | 0 | 0.0 | 0.0 |
| **TM6** | 0 | 0 | 0 | 0.0 | 0.1 | 0.1 | 0.1 | 0 | 0 | 0.0 | 0.0 |
| **Fusobacteria** | 0 | 0 | 0.1 | 0.0 | 0 | 0 | 0.0 | 0 | 0 | 0.0 | 0.0 |
| **Other** | 0 | 0.1 | 0 | 0.0 | 0 | 0 | 0.0 | 0 | 0 | 0.0 | 0.0 |
| **Deinococcus-Thermus** | 0 | 0 | 0.1 | 0.0 | 0 | 0 | 0.0 | 0 | 0 | 0.0 | 0.0 |
| **KB1** | 0 | 0 | 0.1 | 0.0 | 0 | 0 | 0.0 | 0 | 0 | 0.0 | 0.0 |
| **SHA-109** | 0 | 0 | 0 | 0.0 | 0 | 0 | 0.0 | 0 | 0 | 0.0 | 0.0 |
| **BHI80-139** | 0 | 0 | 0 | 0.0 | 0 | 0 | 0.0 | 0 | 0 | 0.0 | 0.0 |
| **OP8** | 0 | 0 | 0 | 0.0 | 0 | 0 | 0.0 | 0 | 0.1 | 0.1 | 0.0 |
| **Caldiserica** | 0 | 0 | 0 | 0.0 | 0 | 0 | 0.0 | 0 | 0.1 | 0.1 | 0.0 |
| **OC31** | 0 | 0 | 0 | 0.0 | 0 | 0 | 0.0 | 0 | 0 | 0.0 | 0.0 |
| **Thermotogae** | 0 | 0 | 0 | 0.0 | 0 | 0 | 0.0 | 0 | 0 | 0.0 | 0.0 |
| **GOUTA4** | 0 | 0 | 0 | 0.0 | 0 | 0 | 0.0 | 0 | 0 | 0.0 | 0.0 |
