# Supplementary material for: Geochemical Influence on Microbial Communities at CO2-Leakage Analog Sites
Source: Front Microbiol. 2017 Nov 9;8:2203. doi: 10.3389/fmicb.2017.02203 (PMC5684959; doi:10.3389/fmicb.2017.02203)
Supplement: Supplementary file 4 [file Table4.DOCX]

S4 Table. Taxonomic coverage of two primers evaluated by the Probe Match tool in RDP^a^

| **Taxonomic group** | **Coverage of primer:** | | | |
| --- | --- | --- | --- | --- |
|  | **341F** | **805R** | **515F** | **805R'** |
| **Domain_Bacteria** | **0.95** | **0.90** | **0.93** | **0.91** |
| Actinobacteria | 0.97 | 0.72 | 0.73 | 0.72 |
| Aquificae | 0.97 | 0.95 | 0.97 | 0.96 |
| Bacteroidetes | 0.90 | 0.96 | 0.97 | 0.97 |
| Caldiserica | 0.98 | 0.96 | 0.01 | 0.96 |
| Chlamydiae | 0.79 | 0.97 | 0.00 | 0.97 |
| Chlorobi | 0.93 | 0.96 | 0.01 | 0.97 |
| Chloroflexi | 0.89 | 0.36 | 0.96 | 0.37 |
| Chrysiogenetes | 0.85 | 1.00 | 1.00 | 1.00 |
| Deferribacteres | 0.99 | 0.96 | 0.98 | 0.97 |
| Deinococcus-Thermus | 0.97 | 0.97 | 0.98 | 0.97 |
| Dictyoglomi | 1.00 | 1.00 | 0.97 | 1.00 |
| Elusimicrobia | 0.84 | 0.96 | 0.98 | 0.97 |
| Fibrobacteres | 0.96 | 0.95 | 0.96 | 0.96 |
| Fusobacteria | 0.95 | 0.97 | 0.97 | 0.98 |
| Gemmatimonadetes | 0.98 | 0.94 | 0.97 | 0.94 |
| Lentisphaerae | 0.93 | 0.96 | 0.97 | 0.96 |
| Nitrospirae | 0.98 | 0.95 | 0.97 | 0.96 |
| Planctomycetes | 0.80 | 0.93 | 0.94 | 0.94 |
| Proteobacteria | 0.97 | 0.93 | 0.97 | 0.94 |
| Spirochaetes | 0.92 | 0.82 | 0.97 | 0.87 |
| Synergistetes | 0.98 | 0.94 | 0.97 | 0.94 |
| Tenericutes | 0.95 | 0.96 | 0.94 | 0.96 |
| Thermodesulfobacteria | 0.96 | 0.98 | 0.96 | 0.99 |
| Thermotogae | 0.97 | 0.95 | 0.97 | 0.96 |
| BRC1 | 0.94 | 0.97 | 0.97 | 0.97 |
| Parcubacteria | 0.65 | 0.88 | 0.00 | 0.89 |
| Microgenomates | 0.21 | 0.00 | 0.24 | 0.00 |
| SR1 | 0.95 | 0.94 | 0.96 | 0.98 |
| Candidatus Saccharibacteria | 0.96 | 0.89 | 0.01 | 0.89 |
| Latescibacteria | 0.96 | 0.97 | 0.97 | 0.97 |
| Armatimonadetes | 0.32 | 0.91 | 0.97 | 0.91 |
| Verrucomicrobia | 0.98 | 0.92 | 0.96 | 0.93 |
| Acidobacteria | 0.97 | 0.95 | 0.98 | 0.95 |
| Firmicutes | 0.96 | 0.96 | 0.97 | 0.96 |
| Cyanobacteria/Chloroplast | 0.92 | 0.93 | 0.97 | 0.95 |
| Marinimicrobia | 0.93 | 0.84 | 0.98 | 0.84 |
| Aminicenantes | 0.97 | 0.97 | 0.98 | 0.97 |
| Omnitrophica | 1.00 | 0.82 | 0.97 | 0.85 |
| Acetothermia | 0.92 | 0.83 | 1.00 | 0.83 |
| Poribacteria | 0.73 | 0.61 | 0.38 | 0.61 |
| Atribacteria | 0.99 | 0.99 | 0.99 | 0.99 |
| Cloacimonetes | 0.98 | 0.91 | 0.97 | 0.91 |
| Candidatus Calescamantes | 0.67 | 0.00 | 1.00 | 0.00 |
| candidate division WPS-1 | 0.97 | 0.95 | 0.97 | 0.96 |
| candidate division WPS-2 | 0.98 | 0.93 | 0.98 | 0.94 |
| Hydrogenedentes | 0.96 | 0.94 | 0.97 | 0.95 |
| candidate division ZB3 | 0.98 | 0.96 | 1.00 | 0.96 |
| Ignavibacteriae | 0.95 | 0.94 | 0.97 | 0.94 |
| Nitrospinae | 0.99 | 0.59 | 0.99 | 0.59 |
| **Domain_Archaea** | **0.00** | **0.94** | **0.56** | **0.95** |
| Crenarchaeota | 0.00 | 0.89 | 0.02 | 0.89 |
| Euryarchaeota | 0.00 | 0.96 | 0.95 | 0.96 |
| Korarchaeota | 0.00 | 0.93 | 0.31 | 0.94 |
| Nanoarchaeota | 0.00 | 0.00 | 0.00 | 0.00 |
| Thaumarchaeota | 0.00 | 0.96 | 0.00 | 0.97 |
| Nanohaloarchaeota | 0.00 | 0.22 | 0.00 | 0.31 |
| Woesearchaeota | 0.00 | 0.92 | 0.97 | 0.93 |
| Pacearchaeota | 0.00 | 0.50 | 1.00 | 0.50 |
| Aigarchaeota | 0.00 | 0.00 | 0.00 | 0.00 |
| Diapherotrites | 0.00 | 0.92 | 0.04 | 0.93 |
| Aenigmarchaeota | 0.00 | 0.86 | 0.86 | 0.86 |
| Parvarchaeota | 0.00 | 0.00 | 0.00 | 0.00 |

RDP’s probe match was conducted against release 11, update 5, of RDP, with sequences data in the specified *E. coli* region (GenBank J01695) for Bacteria or Archaea with good-quality scores that span *E. coli* positions 300 to 850. Primer sequences were the following: 341F=CCTACGGGNGGCWGCAG; 805R= GACTACHVGGGTATCTAATCC, CCTAHGGGRBGCAGCAG; 515F= GTGCCAGCMGCCGCGGTAA, 805Rʹ, TACHVGGGTATCTAATCC.
