# Supplementary material for: Geochemical Influence on Microbial Communities at CO2-Leakage Analog Sites
Source: Front Microbiol. 2017 Nov 9;8:2203. doi: 10.3389/fmicb.2017.02203 (PMC5684959; doi:10.3389/fmicb.2017.02203)
Supplement: Supplementary file 5 [file Table5.DOCX]

S5 Table. Top 100 bacterial genera used for NMDS plot

| **No.** | **#OTU ID** | **DPS2** | **DPW1** | **DPW2** | **DPW6** | **DPW7** | **DPW8** | **BG** |
| --- | --- | --- | --- | --- | --- | --- | --- | --- |
| 1 | **P__Proteobacteria;C__Gammaproteobacteria;O__Pseudomonadales;F__Pseudomonadaceae;G__Pseudomonas** | 0.2 | 0.0 | **27.0** | **4.2** | **5.9** | **35.3** | **9.5** |
| 2 | **Unassigned;Other;Other;Other;Other;Other** | **8.8** | **6.7** | **5.9** | **16.8** | **16.6** | **11.8** | **13.5** |
| 3 | **P__Candidate division OD1;C__uncultured bacterium;O__;F__;G__** | **8.3** | **2.1** | **1.5** | **8.9** | **6.3** | **6.8** | **7.1** |
| 4 | **P__Candidate division TM7;C__uncultured bacterium;O__;F__;G__** | 0.2 | **29.5** | **1.5** | **2.5** | **2.3** | 0.1 | 0.0 |
| 5 | **P__Proteobacteria;C__Betaproteobacteria;O__Burkholderiales;F__Oxalobacteraceae;G__Massilia** | **1.0** | 0.0 | 0.0 | 0.1 | **25.5** | 0.3 | 0.9 |
| 6 | **P__Proteobacteria;C__Alphaproteobacteria;O__Sphingomonadales;F__Sphingomonadaceae;G__Sphingomonas** | **3.9** | **6.6** | **3.2** | 0.4 | 0.1 | 0.0 | **1.4** |
| 7 | **P__Nitrospirae;C__Nitrospira;O__Nitrospirales;F__Nitrospiraceae;G__uncultured** | 0.0 | 0.0 | 0.0 | 0.0 | 0.0 | **8.8** | **6.0** |
| 8 | **P__Proteobacteria;C__Betaproteobacteria;O__Burkholderiales;F__Comamonadaceae;G__Simplicispira** | **5.6** | **6.0** | **1.5** | 0.0 | 0.0 | 0.0 | **0.9** |
| 9 | **P__Actinobacteria;C__Actinobacteria;O__Corynebacteriales;F__Nocardiaceae;G__Rhodococcus** | 0.0 | **9.0** | **0.9** | 0.7 | 0.1 | 0.1 | **3.0** |
| 10 | **P__Cyanobacteria;C__Chloroplast;Other;Other;Other** | **2.3** | 0.1 | **6.6** | 0.1 | 0.1 | 0.0 | **1.4** |
| 11 | **P__Actinobacteria;C__Actinobacteria;O__Micrococcales;F__Micrococcaceae;G__Arthrobacter** | 0.0 | **1.1** | 0.5 | **2.4** | 0.1 | 0.1 | **5.8** |
| 12 | **P__Bacteroidetes;C__Sphingobacteriia;O__Sphingobacteriales;F__Chitinophagaceae;G__Sediminibacterium** | **4.5** | 0.8 | **2.8** | **1.4** | 0.0 | 0.0 | 0.4 |
| 13 | **P__Proteobacteria;C__Betaproteobacteria;O__Hydrogenophilales;F__Hydrogenophilaceae;G__uncultured** | 0.0 | 0.0 | 0.0 | 0.0 | 0.0 | **8.1** | **1.5** |
| 14 | **P__Bacteroidetes;C__Flavobacteriia;O__Flavobacteriales;F__Flavobacteriaceae;G__Flavobacterium** | **3.6** | 0.0 | 0.1 | 0.1 | **2.5** | **1.5** | 0.0 |
| 15 | **P__Proteobacteria;C__Betaproteobacteria;O__Burkholderiales;F__Comamonadaceae;Other** | **4.3** | **0.9** | 0.4 | 0.6 | 0.3 | 0.5 | 0.8 |
| 16 | **P__Proteobacteria;C__Betaproteobacteria;O__Nitrosomonadales;F__Gallionellaceae;G__Gallionella** | 0.0 | 0.0 | 0.1 | **6.6** | 0.0 | 0.1 | 0.4 |
| 17 | **P__Candidate division OD1;Other;Other;Other;Other** | 0.4 | 0.1 | 0.4 | **2.6** | **1.3** | **1.8** | 0.7 |
| 18 | **P__Proteobacteria;C__Alphaproteobacteria;O__Rhizobiales;F__Rhizobiaceae;G__Rhizobium** | **3.9** | **0.9** | 0.8 | 0.1 | 0.0 | 0.0 | **1.0** |
| 19 | **P__Bacteroidetes;C__Cytophagia;O__Cytophagales;F__Cytophagaceae;G__Arcicella** | **2.5** | 0.0 | 0.5 | 0.0 | 0.3 | 0.1 | **3.1** |
| 20 | **P__Proteobacteria;C__Betaproteobacteria;O__Burkholderiales;F__Oxalobacteraceae;Other** | **2.1** | 0.2 | 0.6 | **1.8** | **0.9** | 0.0 | 0.8 |
| 21 | **P__Chloroflexi;C__Anaerolineae;O__Anaerolineales;F__Anaerolineaceae;G__uncultured** | 0.0 | 0.0 | 0.2 | 0.2 | 0.2 | **2.2** | **3.6** |
| 22 | **P__Proteobacteria;C__Betaproteobacteria;O__Nitrosomonadales;F__Gallionellaceae;Other** | 0.0 | 0.0 | 0.2 | **2.9** | 0.0 | **1.5** | **1.4** |
| 23 | **P__Actinobacteria;C__Thermoleophilia;O__Gaiellales;F__uncultured;G__uncultured bacterium** | 0.0 | 0.0 | 0.1 | 0.1 | 0.1 | 0.1 | **5.6** |
| 24 | **P__Proteobacteria;C__Gammaproteobacteria;O__Xanthomonadales;F__Xanthomonadaceae;G__Rhodanobacter** | 0.0 | **5.4** | 0.3 | 0.0 | 0.0 | 0.0 | 0.1 |
| 25 | **P__Proteobacteria;C__Betaproteobacteria;O__Burkholderiales;F__Comamonadaceae;G__uncultured** | **3.8** | 0.6 | 0.2 | 0.2 | 0.5 | 0.0 | 0.5 |
| 26 | **P__BD1-5;C__uncultured bacterium;O__;F__;G__** | 0.3 | 0.1 | 0.0 | **2.7** | **1.3** | 0.5 | 0.6 |
| 27 | **P__Proteobacteria;C__Betaproteobacteria;O__Burkholderiales;F__Comamonadaceae;G__Rhizobacter** | 0.2 | 0.2 | **5.0** | 0.0 | 0.0 | 0.0 | 0.0 |
| 28 | **P__Proteobacteria;C__Alphaproteobacteria;O__Sphingomonadales;F__Sphingomonadaceae;Other** | **1.8** | **2.9** | 0.1 | 0.1 | 0.2 | 0.0 | 0.1 |
| 29 | **P__Bacteroidetes;C__Sphingobacteriia;O__Sphingobacteriales;F__Chitinophagaceae;G__uncultured** | **1.5** | **2.0** | 0.2 | **1.0** | 0.1 | 0.0 | 0.2 |
| 30 | **P__WD272;C__uncultured bacterium;O__;F__;G__** | 0.0 | 0.0 | **4.8** | 0.0 | 0.1 | 0.0 | 0.1 |
| 31 | **P__Candidate division OD1;C__uncultured soil bacterium;O__;F__;G__** | **1.9** | 0.2 | 0.2 | **1.5** | 0.5 | 0.1 | 0.3 |
| 32 | **P__Firmicutes;C__Clostridia;O__Clostridiales;F__Clostridiaceae 1;G__Clostridium sensu stricto 1** | **3.6** | 0.0 | 0.5 | 0.1 | 0.0 | 0.0 | 0.3 |
| 33 | **P__Bacteroidetes;C__Sphingobacteriia;O__Sphingobacteriales;F__Sphingobacteriaceae;G__Mucilaginibacter** | **3.3** | 0.6 | 0.3 | 0.0 | 0.1 | 0.0 | 0.3 |
| 34 | **P__Bacteroidetes;C__Sphingobacteriia;O__Sphingobacteriales;F__env.OPS 17;G__uncultured Bacteroidetes bacterium** | **2.8** | 0.5 | 0.0 | 0.3 | 0.2 | 0.0 | 0.8 |
| 35 | **P__Candidate division OP11;C__uncultured bacterium;O__;F__;G__** | 0.0 | 0.2 | **1.8** | 0.6 | 0.3 | **1.2** | 0.1 |
| 36 | **P__Cyanobacteria;C__Chloroplast;O__uncultured bacterium;F__;G__** | **2.4** | 0.0 | 0.4 | 0.1 | 0.3 | 0.0 | 0.8 |
| 37 | **P__Bacteroidetes;C__Sphingobacteriia;O__Sphingobacteriales;F__PHOS-HE51;G__uncultured bacterium** | 0.0 | 0.1 | **1.5** | 0.4 | **1.7** | 0.2 | 0.1 |
| 38 | **P__Actinobacteria;C__Actinobacteria;O__Corynebacteriales;F__Mycobacteriaceae;G__Mycobacterium** | 0.0 | 0.7 | 0.3 | **1.8** | 0.8 | 0.0 | 0.0 |
| 39 | **P__Proteobacteria;C__Betaproteobacteria;O__Burkholderiales;F__Comamonadaceae;G__Delftia** | 0.0 | 0.0 | **1.9** | 0.0 | 0.0 | 0.3 | **1.3** |
| 40 | **P__Bacteroidetes;C__Sphingobacteriia;O__Sphingobacteriales;F__FFCH9454;G__uncultured bacterium** | 0.0 | 0.0 | 0.0 | 0.0 | **3.5** | 0.0 | 0.0 |
| 41 | **P__Bacteroidetes;C__Sphingobacteriia;O__Sphingobacteriales;F__env.OPS 17;G__uncultured bacterium** | 0.3 | **1.0** | 0.1 | 0.6 | **1.1** | 0.0 | 0.1 |
| 42 | **P__Proteobacteria;C__Gammaproteobacteria;O__Pseudomonadales;F__Moraxellaceae;G__Alkanindiges** | **2.4** | 0.2 | 0.4 | 0.0 | 0.0 | 0.0 | 0.1 |
| 43 | **P__Candidate division OP3;C__uncultured bacterium;O__;F__;G__** | 0.0 | 0.0 | 0.0 | 0.2 | **1.1** | 0.7 | **1.0** |
| 44 | **P__Firmicutes;C__Negativicutes;O__Selenomonadales;F__Veillonellaceae;G__Pelosinus** | **3.0** | 0.0 | 0.0 | 0.0 | 0.0 | 0.0 | 0.0 |
| 45 | **P__Proteobacteria;C__Betaproteobacteria;O__Nitrosomonadales;F__Gallionellaceae;G__Sideroxydans** | 0.0 | 0.0 | 0.0 | **2.1** | 0.0 | 0.0 | 0.7 |
| 46 | **P__Proteobacteria;C__Alphaproteobacteria;O__Sphingomonadales;F__Sphingomonadaceae;G__Novosphingobium** | **1.1** | **1.2** | 0.1 | 0.1 | 0.1 | 0.0 | 0.3 |
| 47 | **P__Bacteroidetes;C__Sphingobacteriia;O__Sphingobacteriales;F__Chitinophagaceae;G__Ferruginibacter** | **1.4** | 0.7 | 0.2 | 0.0 | 0.1 | 0.0 | 0.4 |
| 48 | **P__Nitrospirae;C__Nitrospira;O__Nitrospirales;F__Nitrospiraceae;G__Nitrospira** | 0.0 | 0.1 | **1.0** | **1.3** | 0.2 | 0.0 | 0.0 |
| 49 | **P__Firmicutes;C__Clostridia;O__Clostridiales;F__Clostridiaceae 1;G__Clostridium sensu stricto 9** | **2.5** | 0.0 | 0.0 | 0.0 | 0.0 | 0.0 | 0.0 |
| 50 | **P__Chlorobi;C__Ignavibacteria;O__Ignavibacteriales;F__BSV40;G__uncultured bacterium** | 0.0 | 0.0 | 0.0 | 0.0 | 0.0 | 0.4 | **2.2** |
| 51 | **P__Bacteroidetes;C__Sphingobacteriia;O__Sphingobacteriales;F__Sphingobacteriaceae;G__Pedobacter** | 0.7 | 0.3 | 0.2 | 0.3 | 0.8 | 0.0 | 0.3 |
| 52 | **P__Nitrospirae;C__Nitrospira;O__Nitrospirales;F__4-29;G__uncultured bacterium** | 0.0 | 0.0 | 0.0 | 0.0 | 0.1 | 0.2 | **2.1** |
| 53 | **P__Elusimicrobia;C__Elusimicrobia;O__Lineage IV;F__uncultured bacterium;G__** | 0.0 | 0.0 | 0.4 | 0.6 | 0.4 | 0.5 | 0.2 |
| 54 | **P__Proteobacteria;C__Betaproteobacteria;O__Nitrosomonadales;F__Gallionellaceae;G__uncultured** | 0.0 | 0.0 | 0.2 | **1.8** | 0.0 | 0.1 | 0.1 |
| 55 | **P__Chlorobi;C__Ignavibacteria;O__Ignavibacteriales;F__LD-RB-34;G__uncultured bacterium** | 0.0 | 0.0 | 0.0 | 0.0 | 0.0 | **1.9** | 0.0 |
| 56 | **P__Bacteroidetes;C__Sphingobacteriia;O__Sphingobacteriales;F__KD3-93;G__uncultured bacterium** | 0.0 | **1.2** | 0.1 | 0.5 | 0.1 | 0.0 | 0.0 |
| 57 | **P__Planctomycetes;C__Planctomycetacia;O__Planctomycetales;F__Planctomycetaceae;G__uncultured** | 0.0 | 0.0 | 0.0 | **1.0** | 0.6 | 0.0 | 0.0 |
| 58 | **P__SM2F11;C__uncultured bacterium;O__;F__;G__** | 0.2 | 0.1 | 0.6 | 0.6 | 0.3 | 0.0 | 0.0 |
| 59 | **P__Planctomycetes;C__Planctomycetacia;O__Planctomycetales;F__Planctomycetaceae;G__Planctomyces** | 0.0 | 0.0 | 0.0 | 0.5 | **1.2** | 0.0 | 0.0 |
| 60 | **P__Chloroflexi;C__uncultured;O__uncultured bacterium;F__;G__** | 0.0 | 0.0 | 0.1 | 0.2 | 0.1 | 0.0 | **1.2** |
| 61 | **P__Proteobacteria;C__Alphaproteobacteria;O__Caulobacterales;F__Caulobacteraceae;G__Brevundimonas** | 0.1 | 0.0 | 0.8 | 0.1 | 0.3 | 0.1 | 0.1 |
| 62 | **P__Proteobacteria;C__Deltaproteobacteria;O__Bdellovibrionales;F__Bdellovibrionaceae;G__Bdellovibrio** | 0.1 | 0.2 | 0.1 | 0.5 | 0.5 | 0.0 | 0.0 |
| 63 | **P__Proteobacteria;C__Alphaproteobacteria;O__Rhodobacterales;F__Rhodobacteraceae;Other** | 0.0 | 0.0 | 0.0 | 0.0 | 0.0 | **1.2** | 0.1 |
| 64 | **P__Proteobacteria;C__Betaproteobacteria;O__Burkholderiales;F__Oxalobacteraceae;G__Undibacterium** | 0.2 | 0.1 | 0.3 | 0.0 | 0.5 | 0.0 | 0.2 |
| 65 | **P__Cyanobacteria;C__Cyanobacteria;O__SubsectionIII;F__FamilyI;G__uncultured** | 0.0 | 0.0 | **1.3** | 0.0 | 0.0 | 0.0 | 0.0 |
| 66 | **P__Actinobacteria;C__Actinobacteria;O__Micrococcales;F__Micrococcaceae;Other** | 0.0 | **1.2** | 0.0 | 0.0 | 0.0 | 0.0 | 0.0 |
| 67 | **P__Proteobacteria;C__Betaproteobacteria;O__Burkholderiales;F__Oxalobacteraceae;G__Janthinobacterium** | 0.4 | 0.0 | 0.0 | 0.0 | 0.8 | 0.0 | 0.0 |
| 68 | **P__Nitrospirae;C__Nitrospira;O__Nitrospirales;F__0319-6A21;G__uncultured bacterium** | 0.0 | 0.0 | 0.1 | 0.1 | **1.0** | 0.0 | 0.0 |
| 69 | **P__Firmicutes;C__Bacilli;O__Lactobacillales;F__Streptococcaceae;G__Lactococcus** | 0.0 | **1.2** | 0.0 | 0.0 | 0.0 | 0.0 | 0.0 |
| 70 | **P__Candidate division TM7;Other;Other;Other;Other** | 0.0 | 0.7 | 0.3 | 0.2 | 0.0 | 0.0 | 0.0 |
| 71 | **P__Candidate division OD1;C__uncultured Parcubacteria bacterium;O__;F__;G__** | 0.0 | 0.0 | 0.0 | 0.4 | 0.4 | 0.3 | 0.1 |
| 72 | **P__Proteobacteria;C__Betaproteobacteria;O__Burkholderiales;F__Comamonadaceae;G__Variovorax** | 0.5 | 0.1 | 0.1 | 0.3 | 0.0 | 0.0 | 0.2 |
| 73 | **P__Proteobacteria;C__Gammaproteobacteria;O__Xanthomonadales;F__Xanthomonadaceae;G__Dokdonella** | 0.0 | **1.2** | 0.0 | 0.0 | 0.0 | 0.0 | 0.0 |
| 74 | **P__Firmicutes;C__Bacilli;O__Lactobacillales;F__Leuconostocaceae;G__Leuconostoc** | 0.0 | **1.2** | 0.0 | 0.0 | 0.0 | 0.0 | 0.0 |
| 75 | **P__Nitrospirae;C__Nitrospira;O__Nitrospirales;F__Nitrospiraceae;G__uncultured bacterium** | 0.0 | 0.0 | 0.0 | 0.0 | 0.0 | 0.3 | **0.9** |
| 76 | **P__Bacteroidetes;C__Bacteroidia;O__Bacteroidales;F__Bacteroidaceae;G__Bacteroides** | 0.0 | 0.0 | **0.9** | 0.0 | 0.0 | 0.0 | 0.1 |
| 77 | **P__Bacteroidetes;C__Flavobacteriia;O__Flavobacteriales;F__Flavobacteriaceae;G__Chryseobacterium** | 0.0 | 0.8 | 0.3 | 0.0 | 0.0 | 0.0 | 0.0 |
| 78 | **P__Bacteroidetes;C__Cytophagia;O__Cytophagales;F__Cytophagaceae;G__uncultured** | 0.0 | 0.0 | 0.0 | **1.0** | 0.0 | 0.0 | 0.0 |
| 79 | **P__Proteobacteria;C__Alphaproteobacteria;O__Caulobacterales;F__Caulobacteraceae;G__Phenylobacterium** | 0.4 | 0.1 | 0.1 | 0.2 | 0.3 | 0.0 | 0.1 |
| 80 | **P__Actinobacteria;C__Coriobacteriia;O__Coriobacteriales;F__Coriobacteriaceae;G__uncultured** | 0.0 | 0.0 | 0.0 | 0.0 | 0.0 | **1.0** | 0.0 |
| 81 | **P__Proteobacteria;C__Alphaproteobacteria;O__Rhizobiales;F__Bradyrhizobiaceae;G__Nitrobacter** | 0.1 | 0.8 | 0.0 | 0.1 | 0.1 | 0.0 | 0.0 |
| 82 | **P__Acidobacteria;C__Holophagae;O__Holophagales;F__Holophagaceae;G__uncultured** | 0.1 | 0.1 | 0.7 | 0.0 | 0.0 | 0.0 | 0.0 |
| 83 | **P__Proteobacteria;C__Epsilonproteobacteria;O__Campylobacterales;F__Campylobacteraceae;G__Campylobacter** | 0.0 | 0.0 | 0.7 | 0.0 | 0.0 | 0.0 | 0.3 |
| 84 | **P__TA06;C__uncultured bacterium;O__;F__;G__** | 0.0 | 0.0 | 0.0 | 0.1 | 0.0 | 0.0 | **0.9** |
| 85 | **P__Chlamydiae;C__Chlamydiae;O__Chlamydiales;F__Simkaniaceae;G__Candidatus Rhabdochlamydia** | 0.0 | 0.0 | 0.0 | 0.8 | 0.2 | 0.0 | 0.0 |
| 86 | **P__Chlorobi;C__Ignavibacteria;O__Ignavibacteriales;F__PHOS-HE36;G__uncultured bacterium** | 0.0 | 0.0 | 0.0 | 0.0 | 0.0 | **0.9** | 0.0 |
| 87 | **P__Proteobacteria;C__Alphaproteobacteria;O__Rhizobiales;F__Beijerinckiaceae;G__uncultured** | 0.0 | 0.1 | 0.8 | 0.0 | 0.0 | 0.0 | 0.0 |
| 88 | **P__Proteobacteria;C__Alphaproteobacteria;O__Rhodobacterales;F__Rhodobacteraceae;G__Rhodobacter** | 0.7 | 0.0 | 0.1 | 0.1 | 0.0 | 0.0 | 0.1 |
| 89 | **P__Proteobacteria;C__Deltaproteobacteria;O__Desulfovibrionales;F__Desulfovibrionaceae;G__Desulfovibrio** | 0.0 | 0.0 | 0.0 | 0.0 | 0.0 | **0.9** | 0.0 |
| 90 | **P__Actinobacteria;C__Actinobacteria;O__Micrococcales;F__Intrasporangiaceae;G__Lapillicoccus** | 0.0 | **0.9** | 0.0 | 0.0 | 0.0 | 0.0 | 0.0 |
| 91 | **P__Candidate division WS6;C__uncultured bacterium;O__;F__;G__** | 0.0 | 0.0 | 0.0 | 0.1 | 0.8 | 0.0 | 0.0 |
| 92 | **P__Verrucomicrobia;C__OPB35 soil group;O__uncultured bacterium;F__;G__** | 0.0 | 0.0 | 0.1 | 0.3 | 0.3 | 0.0 | 0.1 |
| 93 | **P__Proteobacteria;C__Deltaproteobacteria;O__Desulfobacterales;F__Nitrospinaceae;G__uncultured** | 0.0 | 0.0 | 0.0 | 0.0 | 0.0 | 0.7 | 0.1 |
| 94 | **P__Proteobacteria;C__Alphaproteobacteria;O__Rhizobiales;F__Methylobacteriaceae;G__Methylobacterium** | 0.0 | 0.4 | 0.3 | 0.0 | 0.0 | 0.1 | 0.0 |
| 95 | **P__Proteobacteria;C__Betaproteobacteria;O__Burkholderiales;F__Comamonadaceae;G__Acidovorax** | 0.1 | 0.2 | 0.2 | 0.0 | 0.1 | 0.1 | 0.2 |
| 96 | **P__Armatimonadetes;C__Chthonomonadetes;O__Chthonomonadales;F__Chthonomonadaceae;G__Chthonomonas** | 0.0 | 0.0 | 0.8 | 0.0 | 0.0 | 0.0 | 0.0 |
| 97 | **P__Firmicutes;C__Clostridia;O__Clostridiales;F__Clostridiaceae 1;G__Clostridium sensu stricto 2** | 0.0 | 0.0 | 0.0 | 0.8 | 0.0 | 0.0 | 0.0 |
| 98 | **P__Bacteroidetes;C__Sphingobacteriia;O__Sphingobacteriales;F__NS11-12 marine group;G__uncultured bacterium** | 0.5 | 0.0 | 0.0 | 0.1 | 0.1 | 0.0 | 0.1 |
| 99 | **P__Actinobacteria;C__Actinobacteria;O__Corynebacteriales;F__uncultured;Other** | 0.0 | 0.7 | 0.1 | 0.0 | 0.0 | 0.0 | 0.0 |
| 100 | **P__Acidobacteria;C__Acidobacteria;O__Subgroup 11;F__uncultured bacterium;G__** | 0.0 | 0.0 | 0.8 | 0.0 | 0.0 | 0.0 | 0.0 |
