# Supplementary material for: Geochemical Influence on Microbial Communities at CO2-Leakage Analog Sites
Source: Front Microbiol. 2017 Nov 9;8:2203. doi: 10.3389/fmicb.2017.02203 (PMC5684959; doi:10.3389/fmicb.2017.02203)
Supplement: Supplementary file 6 [file Table6.DOCX]

S6 Table. Bacterial community compositions of MiSeq sequencing using 341F-805R (genus level)

| 1. **Firmicutes - #OTU ID** | | **DPS2** | | **DPW1** | | **DPW2** | | **DPW6** | | **DPW7** | | **DPW8** | | **BG** |
| --- | --- | --- | --- | --- | --- | --- | --- | --- | --- | --- | --- | --- | --- | --- |
| **P__Firmicutes;C__Clostridia;O__Clostridiales;F__Clostridiaceae 1;G__Clostridium sensu stricto 1** | | 3.6 | | 0.0 | | 0.5 | | 0.1 | | 0.0 | | 0.0 | | 0.3 |
| **P__Firmicutes;C__Negativicutes;O__Selenomonadales;F__Veillonellaceae;G__Pelosinus** | | 3.0 | | 0.0 | | 0.0 | | 0.0 | | 0.0 | | 0.0 | | 0.0 |
| **P__Firmicutes;C__Clostridia;O__Clostridiales;F__Clostridiaceae 1;G__Clostridium sensu stricto 9** | | 2.5 | | 0.0 | | 0.0 | | 0.0 | | 0.0 | | 0.0 | | 0.0 |
| **P__Firmicutes;C__Bacilli;O__Lactobacillales;F__Streptococcaceae;G__Lactococcus** | | 0.0 | | 1.2 | | 0.0 | | 0.0 | | 0.0 | | 0.0 | | 0.0 |
| **P__Firmicutes;C__Bacilli;O__Lactobacillales;F__Leuconostocaceae;G__Leuconostoc** | | 0.0 | | 1.2 | | 0.0 | | 0.0 | | 0.0 | | 0.0 | | 0.0 |
| **P__Firmicutes;C__Clostridia;O__Clostridiales;F__Clostridiaceae 1;G__Clostridium sensu stricto 2** | | 0.0 | | 0.0 | | 0.0 | | 0.8 | | 0.0 | | 0.0 | | 0.0 |
| **P__Firmicutes;C__Bacilli;O__Lactobacillales;F__Lactobacillaceae;G__Lactobacillus** | | 0.0 | | 0.0 | | 0.0 | | 0.0 | | 0.0 | | 0.1 | | 0.6 |
| **P__Firmicutes;C__Bacilli;O__Lactobacillales;F__Leuconostocaceae;G__Weissella** | | 0.0 | | 0.0 | | 0.3 | | 0.0 | | 0.0 | | 0.2 | | 0.0 |
| **P__Firmicutes;C__Clostridia;O__Clostridiales;F__Clostridiaceae 1;G__Clostridium sensu stricto 12** | | 0.5 | | 0.0 | | 0.0 | | 0.0 | | 0.0 | | 0.0 | | 0.0 |
| **P__Firmicutes;C__Clostridia;O__Clostridiales;F__Lachnospiraceae;G__Roseburia** | | 0.0 | | 0.0 | | 0.5 | | 0.0 | | 0.0 | | 0.0 | | 0.0 |
| **P__Firmicutes;C__Clostridia;O__Clostridiales;F__Clostridiaceae 1;Other** | | 0.2 | | 0.0 | | 0.0 | | 0.1 | | 0.0 | | 0.0 | | 0.0 |
| **P__Firmicutes;C__Bacilli;O__Bacillales;F__Planococcaceae;G__Planomicrobium** | | 0.0 | | 0.0 | | 0.0 | | 0.0 | | 0.0 | | 0.3 | | 0.0 |
| **P__Firmicutes;C__Bacilli;O__Bacillales;F__Alicyclobacillaceae;G__Alicyclobacillus** | | 0.0 | | 0.0 | | 0.3 | | 0.0 | | 0.0 | | 0.0 | | 0.0 |
| **P__Firmicutes;C__Bacilli;O__Bacillales;F__Planococcaceae;G__Paenisporosarcina** | | 0.0 | | 0.0 | | 0.0 | | 0.0 | | 0.0 | | 0.0 | | 0.2 |
| **P__Firmicutes;C__Clostridia;O__Halanaerobiales;F__Halanaerobiaceae;G__Halanaerobium** | | 0.0 | | 0.0 | | 0.2 | | 0.0 | | 0.0 | | 0.0 | | 0.0 |
| **P__Firmicutes;C__Clostridia;O__Clostridiales;F__Ruminococcaceae;G__Faecalibacterium** | | 0.0 | | 0.0 | | 0.0 | | 0.0 | | 0.0 | | 0.1 | | 0.1 |
| **P__Firmicutes;C__Erysipelotrichia;O__Erysipelotrichales;F__Erysipelotrichaceae;G__Incertae Sedis** | | 0.0 | | 0.0 | | 0.2 | | 0.0 | | 0.0 | | 0.0 | | 0.0 |
| **P__Firmicutes;C__Clostridia;O__Clostridiales;F__Clostridiaceae 1;G__Clostridium sensu stricto 13** | | 0.0 | | 0.0 | | 0.2 | | 0.0 | | 0.0 | | 0.0 | | 0.0 |
| **P__Firmicutes;C__Clostridia;O__Clostridiales;F__Lachnospiraceae;G__uncultured** | | 0.0 | | 0.0 | | 0.2 | | 0.0 | | 0.0 | | 0.0 | | 0.0 |
| **P__Firmicutes;C__Clostridia;O__Clostridiales;F__vadinBB60;G__uncultured bacterium** | | 0.0 | | 0.0 | | 0.0 | | 0.0 | | 0.0 | | 0.0 | | 0.1 |
| **P__Firmicutes;C__Clostridia;O__Clostridiales;F__Lachnospiraceae;G__Incertae Sedis** | | 0.0 | | 0.0 | | 0.0 | | 0.0 | | 0.0 | | 0.0 | | 0.1 |
| **P__Firmicutes;C__Bacilli;O__Bacillales;F__Staphylococcaceae;G__Staphylococcus** | | 0.0 | | 0.0 | | 0.0 | | 0.0 | | 0.0 | | 0.0 | | 0.0 |
| **P__Firmicutes;C__Erysipelotrichia;O__Erysipelotrichales;F__Erysipelotrichaceae;G__Turicibacter** | | 0.0 | | 0.0 | | 0.0 | | 0.0 | | 0.0 | | 0.0 | | 0.1 |
| **P__Firmicutes;C__Erysipelotrichia;O__Erysipelotrichales;F__Erysipelotrichaceae;G__uncultured** | | 0.0 | | 0.0 | | 0.0 | | 0.0 | | 0.0 | | 0.0 | | 0.1 |
| **P__Firmicutes;C__Clostridia;O__Clostridiales;F__Ruminococcaceae;G__Incertae Sedis** | | 0.0 | | 0.0 | | 0.0 | | 0.0 | | 0.0 | | 0.0 | | 0.1 |
| **P__Firmicutes;C__Bacilli;O__Lactobacillales;F__Carnobacteriaceae;Other** | | 0.0 | | 0.0 | | 0.0 | | 0.0 | | 0.0 | | 0.1 | | 0.0 |
| **P__Firmicutes;C__Clostridia;O__Clostridiales;F__Peptostreptococcaceae;G__uncultured** | | 0.0 | | 0.0 | | 0.0 | | 0.0 | | 0.0 | | 0.0 | | 0.1 |
| **P__Firmicutes;C__Clostridia;O__Clostridiales;F__Peptococcaceae;G__Desulfitobacterium** | | 0.0 | | 0.0 | | 0.0 | | 0.0 | | 0.0 | | 0.1 | | 0.0 |
| **P__Firmicutes;C__Clostridia;O__Halanaerobiales;F__uncultured;G__uncultured bacterium** | | 0.0 | | 0.0 | | 0.0 | | 0.0 | | 0.0 | | 0.0 | | 0.1 |
| **P__Firmicutes;C__Clostridia;O__Clostridiales;F__Peptococcaceae;G__Desulfurispora** | | 0.0 | | 0.0 | | 0.0 | | 0.0 | | 0.0 | | 0.1 | | 0.0 |
| **P__Firmicutes;C__Clostridia;O__Clostridiales;F__Ruminococcaceae;G__Anaerotruncus** | | 0.0 | | 0.0 | | 0.0 | | 0.0 | | 0.0 | | 0.0 | | 0.1 |
| **P__Firmicutes;C__Clostridia;O__Clostridiales;F__Peptococcaceae;G__Desulfosporosinus** | | 0.0 | | 0.0 | | 0.0 | | 0.0 | | 0.0 | | 0.0 | | 0.0 |
| **P__Firmicutes;C__Clostridia;O__Clostridiales;F__Clostridiaceae 1;G__Clostridium sensu stricto 8** | | 0.0 | | 0.0 | | 0.0 | | 0.0 | | 0.0 | | 0.0 | | 0.0 |
| **Sum** | | **9.8** | | **2.4** | | **2.5** | | **1.2** | | **0.1** | | **1.1** | | **2.0** |
| 1. **Planctomycetes - #OTU ID** | **DPS2** | | **DPW1** | | **DPW2** | | **DPW6** | | **DPW7** | | **DPW8** | | **BG** | |
| **P__Planctomycetes;C__BD7-11;O__uncultured bacterium;F__;G__** | 0.0 | | 0.0 | | 0.0 | | 0.0 | | 0.1 | | 0.0 | | 0.0 | |
| **P__Planctomycetes;C__BD7-11;O__uncultured planctomycete;F__;G__** | 0.0 | | 0.0 | | 0.0 | | 0.0 | | 0.0 | | 0.0 | | 0.0 | |
| **P__Planctomycetes;C__OM190;O__uncultured bacterium;F__;G__** | 0.0 | | 0.0 | | 0.0 | | 0.5 | | 0.1 | | 0.0 | | 0.0 | |
| **P__Planctomycetes;C__OM190;Other;Other;Other** | 0.0 | | 0.0 | | 0.0 | | 0.0 | | 0.0 | | 0.0 | | 0.0 | |
| **P__Planctomycetes;C__Phycisphaerae;O__CCM11a;F__uncultured bacterium;G__** | 0.0 | | 0.0 | | 0.0 | | 0.1 | | 0.1 | | 0.0 | | 0.0 | |
| **P__Planctomycetes;C__Phycisphaerae;O__CCM11a;Other;Other** | 0.0 | | 0.0 | | 0.0 | | 0.0 | | 0.0 | | 0.0 | | 0.0 | |
| **P__Planctomycetes;C__Phycisphaerae;O__mle1-8;F__uncultured organism;G__** | 0.0 | | 0.0 | | 0.0 | | 0.2 | | 0.0 | | 0.0 | | 0.0 | |
| **P__Planctomycetes;C__Phycisphaerae;O__MSBL9;F__uncultured bacterium;G__** | 0.0 | | 0.0 | | 0.0 | | 0.0 | | 0.0 | | 0.0 | | 0.0 | |
| **P__Planctomycetes;C__Phycisphaerae;O__Phycisphaerales;F__Phycisphaeraceae;G__AKYG587** | 0.0 | | 0.0 | | 0.0 | | 0.0 | | 0.0 | | 0.0 | | 0.0 | |
| **P__Planctomycetes;C__Phycisphaerae;O__Phycisphaerales;F__Phycisphaeraceae;G__I-8** | 0.0 | | 0.0 | | 0.0 | | 0.0 | | 0.0 | | 0.1 | | 0.0 | |
| **P__Planctomycetes;C__Phycisphaerae;O__Phycisphaerales;F__Phycisphaeraceae;G__Phycisphaera** | 0.0 | | 0.0 | | 0.0 | | 0.0 | | 0.0 | | 0.0 | | 0.0 | |
| **P__Planctomycetes;C__Phycisphaerae;O__Phycisphaerales;F__Phycisphaeraceae;G__SM1A02** | 0.0 | | 0.0 | | 0.0 | | 0.5 | | 0.1 | | 0.0 | | 0.0 | |
| **P__Planctomycetes;C__Phycisphaerae;O__S-70;F__uncultured bacterium;G__** | 0.0 | | 0.0 | | 0.0 | | 0.0 | | 0.0 | | 0.0 | | 0.0 | |
| **P__Planctomycetes;C__Phycisphaerae;O__WD2101 soil group;F__uncultured bacterium;G__** | 0.2 | | 0.0 | | 0.0 | | 0.1 | | 0.0 | | 0.1 | | 0.0 | |
| **P__Planctomycetes;C__Phycisphaerae;O__WD2101 soil group;Other;Other** | 0.0 | | 0.0 | | 0.0 | | 0.1 | | 0.0 | | 0.0 | | 0.0 | |
| **P__Planctomycetes;C__Pla3 lineage;O__uncultured bacterium;F__;G__** | 0.0 | | 0.0 | | 0.0 | | 0.0 | | 0.0 | | 0.0 | | 0.0 | |
| **P__Planctomycetes;C__Pla3 lineage;Other;Other;Other** | 0.0 | | 0.0 | | 0.0 | | 0.0 | | 0.0 | | 0.0 | | 0.0 | |
| **P__Planctomycetes;C__Pla4 lineage;O__uncultured bacterium;F__;G__** | 0.0 | | 0.0 | | 0.0 | | 0.0 | | 0.1 | | 0.0 | | 0.0 | |
| **P__Planctomycetes;C__Planctomycetacia;O__Brocadiales;F__Brocadiaceae;G__Candidatus Brocadia** | 0.0 | | 0.0 | | 0.0 | | 0.0 | | 0.0 | | 0.0 | | 0.0 | |
| **P__Planctomycetes;C__Planctomycetacia;O__Brocadiales;F__Brocadiaceae;G__W4** | 0.0 | | 0.0 | | 0.0 | | 0.0 | | 0.2 | | 0.0 | | 0.0 | |
| **P__Planctomycetes;C__Planctomycetacia;O__Brocadiales;F__Brocadiaceae;Other** | 0.0 | | 0.0 | | 0.0 | | 0.0 | | 0.0 | | 0.0 | | 0.0 | |
| **P__Planctomycetes;C__Planctomycetacia;O__Planctomycetales;F__Planctomycetaceae;G__Gemmata** | 0.0 | | 0.1 | | 0.0 | | 0.2 | | 0.1 | | 0.0 | | 0.0 | |
| **P__Planctomycetes;C__Planctomycetacia;O__Planctomycetales;F__Planctomycetaceae;G__Pir3 lineage** | 0.0 | | 0.0 | | 0.0 | | 0.0 | | 0.0 | | 0.0 | | 0.0 | |
| **P__Planctomycetes;C__Planctomycetacia;O__Planctomycetales;F__Planctomycetaceae;G__Pir4 lineage** | 0.0 | | 0.0 | | 0.0 | | 0.5 | | 0.0 | | 0.0 | | 0.0 | |
| **P__Planctomycetes;C__Planctomycetacia;O__Planctomycetales;F__Planctomycetaceae;G__Pirellula** | 0.0 | | 0.0 | | 0.0 | | 0.1 | | 0.0 | | 0.0 | | 0.0 | |
| **P__Planctomycetes;C__Planctomycetacia;O__Planctomycetales;F__Planctomycetaceae;G__Planctomyces** | 0.0 | | 0.0 | | 0.0 | | 0.5 | | 1.2 | | 0.0 | | 0.0 | |
| **P__Planctomycetes;C__Planctomycetacia;O__Planctomycetales;F__Planctomycetaceae;G__Schlesneria** | 0.0 | | 0.0 | | 0.0 | | 0.2 | | 0.0 | | 0.0 | | 0.0 | |
| **P__Planctomycetes;C__Planctomycetacia;O__Planctomycetales;F__Planctomycetaceae;G__Singulisphaera** | 0.0 | | 0.0 | | 0.0 | | 0.0 | | 0.0 | | 0.0 | | 0.0 | |
| **P__Planctomycetes;C__Planctomycetacia;O__Planctomycetales;F__Planctomycetaceae;G__uncultured** | 0.0 | | 0.0 | | 0.0 | | 1.0 | | 0.6 | | 0.0 | | 0.0 | |
| **P__Planctomycetes;C__Planctomycetacia;O__Planctomycetales;F__Planctomycetaceae;Other** | 0.0 | | 0.0 | | 0.0 | | 0.0 | | 0.0 | | 0.0 | | 0.0 | |
| **P__Planctomycetes;C__vadinHA49;O__uncultured bacterium;F__;G__** | 0.0 | | 0.0 | | 0.0 | | 0.1 | | 0.2 | | 0.0 | | 0.1 | |
| **Sum** | **0.3** | | **0.2** | | **0.0** | | **4.3** | | **3.0** | | **0.2** | | **0.2** | |

| 1. **Nitrospirae - #OTU ID** | **DPS2** | **DPW1** | **DPW2** | **DPW6** | **DPW7** | **DPW8** | **BG** |
| --- | --- | --- | --- | --- | --- | --- | --- |
| **P__Nitrospirae;C__Nitrospira;O__Nitrospirales;F__0319-6A21;G__uncultured bacterium** | 0.0 | 0.0 | 0.1 | 0.1 | 1.0 | 0.0 | 0.0 |
| **P__Nitrospirae;C__Nitrospira;O__Nitrospirales;F__4-29;G__uncultured bacterium** | 0.0 | 0.0 | 0.0 | 0.0 | 0.1 | 0.2 | 2.1 |
| **P__Nitrospirae;C__Nitrospira;O__Nitrospirales;F__BT44;G__uncultured bacterium** | 0.0 | 0.0 | 0.0 | 0.0 | 0.0 | 0.0 | 0.1 |
| **P__Nitrospirae;C__Nitrospira;O__Nitrospirales;F__MIZ17;G__uncultured bacterium** | 0.0 | 0.0 | 0.0 | 0.0 | 0.0 | 0.0 | 0.0 |
| **P__Nitrospirae;C__Nitrospira;O__Nitrospirales;F__Nitrospiraceae;G__Leptospirillum** | 0.0 | 0.0 | 0.1 | 0.1 | 0.3 | 0.0 | 0.0 |
| **P__Nitrospirae;C__Nitrospira;O__Nitrospirales;F__Nitrospiraceae;G__Nitrospira** | 0.0 | 0.1 | 1.0 | 1.3 | 0.2 | 0.0 | 0.0 |
| **P__Nitrospirae;C__Nitrospira;O__Nitrospirales;F__Nitrospiraceae;G__uncultured** | 0.0 | 0.0 | 0.0 | 0.0 | 0.0 | 8.8 | 6.0 |
| **P__Nitrospirae;C__Nitrospira;O__Nitrospirales;F__Nitrospiraceae;G__uncultured bacterium** | 0.0 | 0.0 | 0.0 | 0.0 | 0.0 | 0.3 | 0.9 |
| **P__Nitrospirae;C__Nitrospira;O__Nitrospirales;F__Nitrospirales Incertae Sedis;G__Candidatus Methylomirabilis** | 0.0 | 0.0 | 0.0 | 0.0 | 0.1 | 0.0 | 0.0 |
| **P__Nitrospirae;C__Nitrospira;O__Nitrospirales;F__Sh765B-TzT-35;G__uncultured bacterium** | 0.0 | 0.0 | 0.0 | 0.0 | 0.1 | 0.0 | 0.0 |
| **P__Nitrospirae;C__Nitrospira;O__Nitrospirales;F__wb1-A12;Other** | 0.0 | 0.0 | 0.0 | 0.0 | 0.0 | 0.0 | 0.0 |
| **Sum** | **0.0** | **0.2** | **1.2** | **1.6** | **1.8** | **9.3** | **9.1** |

| 1. **Chloroflexi - #OTU ID** | **DPS2** | **DPW1** | **DPW2** | **DPW6** | **DPW7** | **DPW8** | **BG** |
| --- | --- | --- | --- | --- | --- | --- | --- |
| **P__Chloroflexi;C__Anaerolineae;O__Anaerolineales;F__Anaerolineaceae;G__Leptolinea** | 0.0 | 0.0 | 0.0 | 0.0 | 0.0 | 0.0 | 0.0 |
| **P__Chloroflexi;C__Anaerolineae;O__Anaerolineales;F__Anaerolineaceae;G__Levilinea** | 0.0 | 0.0 | 0.0 | 0.0 | 0.0 | 0.0 | 0.0 |
| **P__Chloroflexi;C__Anaerolineae;O__Anaerolineales;F__Anaerolineaceae;G__Longilinea** | 0.0 | 0.0 | 0.0 | 0.0 | 0.0 | 0.0 | 0.0 |
| **P__Chloroflexi;C__Anaerolineae;O__Anaerolineales;F__Anaerolineaceae;G__Ornatilinea** | 0.0 | 0.0 | 0.0 | 0.0 | 0.0 | 0.0 | 0.0 |
| **P__Chloroflexi;C__Anaerolineae;O__Anaerolineales;F__Anaerolineaceae;G__uncultured** | 0.0 | 0.0 | 0.2 | 0.2 | 0.2 | 2.2 | 3.6 |
| **P__Chloroflexi;C__Ardenticatenia;O__uncultured;F__uncultured Chloroflexi bacterium;G__** | 0.0 | 0.0 | 0.0 | 0.0 | 0.0 | 0.0 | 0.0 |
| **P__Chloroflexi;C__Caldilineae;O__Caldilineales;F__Caldilineaceae;G__uncultured** | 0.0 | 0.0 | 0.0 | 0.0 | 0.0 | 0.0 | 0.0 |
| **P__Chloroflexi;C__Chloroflexia;O__AKIW781;F__uncultured bacterium;G__** | 0.0 | 0.0 | 0.0 | 0.0 | 0.0 | 0.0 | 0.0 |
| **P__Chloroflexi;C__Chloroflexia;O__AKIW781;Other;Other** | 0.0 | 0.0 | 0.0 | 0.0 | 0.0 | 0.0 | 0.0 |
| **P__Chloroflexi;C__Chloroflexia;O__Chloroflexales;F__Roseiflexaceae;G__Roseiflexus** | 0.0 | 0.0 | 0.0 | 0.0 | 0.0 | 0.0 | 0.0 |
| **P__Chloroflexi;C__Dehalococcoidia;O__GIF3;F__uncultured bacterium;G__** | 0.0 | 0.0 | 0.0 | 0.0 | 0.0 | 0.0 | 0.0 |
| **P__Chloroflexi;C__Dehalococcoidia;O__Sh765B-AG-111;F__uncultured bacterium;G__** | 0.0 | 0.0 | 0.0 | 0.0 | 0.0 | 0.0 | 0.0 |
| **P__Chloroflexi;C__Dehalococcoidia;O__vadinBA26;F__uncultured bacterium;G__** | 0.0 | 0.0 | 0.0 | 0.0 | 0.0 | 0.0 | 0.0 |
| **P__Chloroflexi;C__Dehalococcoidia;Other;Other;Other** | 0.0 | 0.0 | 0.0 | 0.0 | 0.0 | 0.0 | 0.0 |
| **P__Chloroflexi;C__Elev-1554;O__uncultured bacterium;F__;G__** | 0.0 | 0.0 | 0.0 | 0.0 | 0.0 | 0.0 | 0.0 |
| **P__Chloroflexi;C__Gitt-GS-136;O__uncultured bacterium;F__;G__** | 0.0 | 0.0 | 0.0 | 0.0 | 0.0 | 0.0 | 0.0 |
| **P__Chloroflexi;C__JG30-KF-CM66;O__uncultured bacterium;F__;G__** | 0.0 | 0.0 | 0.0 | 0.1 | 0.1 | 0.1 | 0.0 |
| **P__Chloroflexi;C__JG30-KF-CM66;O__uncultured Chloroflexi bacterium;F__;G__** | 0.0 | 0.0 | 0.0 | 0.0 | 0.0 | 0.0 | 0.0 |
| **P__Chloroflexi;C__JG30-KF-CM66;Other;Other;Other** | 0.0 | 0.0 | 0.1 | 0.0 | 0.0 | 0.0 | 0.0 |
| **P__Chloroflexi;C__KD4-96;O__uncultured bacterium;F__;G__** | 0.0 | 0.0 | 0.2 | 0.4 | 0.1 | 0.1 | 0.0 |
| **P__Chloroflexi;C__KD4-96;O__uncultured Chloroflexi bacterium;F__;G__** | 0.0 | 0.0 | 0.0 | 0.0 | 0.0 | 0.0 | 0.0 |
| **P__Chloroflexi;C__KD4-96;Other;Other;Other** | 0.0 | 0.0 | 0.0 | 0.1 | 0.1 | 0.0 | 0.0 |
| **P__Chloroflexi;C__Ktedonobacteria;O__C0119;F__uncultured bacterium;G__** | 0.0 | 0.0 | 0.0 | 0.0 | 0.0 | 0.0 | 0.0 |
| **P__Chloroflexi;C__Ktedonobacteria;O__C0119;F__uncultured Chloroflexi bacterium;G__** | 0.0 | 0.0 | 0.0 | 0.0 | 0.0 | 0.0 | 0.0 |
| **P__Chloroflexi;C__Ktedonobacteria;O__JG30-KF-AS9;F__uncultured bacterium;G__** | 0.0 | 0.0 | 0.0 | 0.0 | 0.0 | 0.0 | 0.0 |
| **P__Chloroflexi;C__Ktedonobacteria;O__Ktedonobacterales;F__G12-WMSP1;G__uncultured Chloroflexi bacterium** | 0.0 | 0.5 | 0.0 | 0.0 | 0.0 | 0.0 | 0.0 |
| **P__Chloroflexi;C__Ktedonobacteria;O__Ktedonobacterales;F__JG30a-KF-32;G__uncultured bacterium** | 0.0 | 0.0 | 0.0 | 0.0 | 0.0 | 0.0 | 0.0 |
| **P__Chloroflexi;C__Ktedonobacteria;O__Ktedonobacterales;F__JG30a-KF-32;Other** | 0.0 | 0.1 | 0.0 | 0.0 | 0.0 | 0.0 | 0.0 |
| **P__Chloroflexi;C__Ktedonobacteria;O__Ktedonobacterales;F__Thermosporotrichaceae;G__uncultured** | 0.0 | 0.0 | 0.1 | 0.0 | 0.0 | 0.0 | 0.0 |
| **P__Chloroflexi;C__P2-11E;O__uncultured bacterium;F__;G__** | 0.0 | 0.0 | 0.1 | 0.0 | 0.2 | 0.0 | 0.0 |
| **P__Chloroflexi;C__S085;O__uncultured bacterium;F__;G__** | 0.0 | 0.0 | 0.0 | 0.0 | 0.1 | 0.0 | 0.1 |
| **P__Chloroflexi;C__S085;O__uncultured Chloroflexi bacterium;F__;G__** | 0.0 | 0.0 | 0.0 | 0.0 | 0.0 | 0.0 | 0.0 |
| **P__Chloroflexi;C__SAR202 clade;O__uncultured bacterium;F__;G__** | 0.0 | 0.0 | 0.0 | 0.4 | 0.1 | 0.0 | 0.0 |
| **P__Chloroflexi;C__SAR202 clade;O__uncultured Chloroflexi bacterium;F__;G__** | 0.0 | 0.0 | 0.0 | 0.0 | 0.0 | 0.0 | 0.0 |
| **P__Chloroflexi;C__SAR202 clade;Other;Other;Other** | 0.0 | 0.0 | 0.0 | 0.0 | 0.0 | 0.0 | 0.0 |
| **P__Chloroflexi;C__Thermomicrobia;O__JG30-KF-CM45;F__uncultured bacterium;G__** | 0.0 | 0.0 | 0.0 | 0.0 | 0.0 | 0.0 | 0.0 |
| **P__Chloroflexi;C__Thermomicrobia;O__JG30-KF-CM45;Other;Other** | 0.0 | 0.0 | 0.0 | 0.0 | 0.0 | 0.0 | 0.0 |
| **P__Chloroflexi;C__TK10;O__uncultured bacterium;F__;G__** | 0.0 | 0.0 | 0.0 | 0.0 | 0.1 | 0.5 | 0.0 |
| **P__Chloroflexi;C__TK10;O__uncultured Chloroflexi bacterium;F__;G__** | 0.0 | 0.0 | 0.0 | 0.0 | 0.0 | 0.0 | 0.0 |
| **P__Chloroflexi;C__TK10;Other;Other;Other** | 0.0 | 0.0 | 0.0 | 0.1 | 0.0 | 0.0 | 0.0 |
| **P__Chloroflexi;C__uncultured Bellilinea sp.;O__;F__;G__** | 0.0 | 0.0 | 0.0 | 0.0 | 0.0 | 0.0 | 0.0 |
| **P__Chloroflexi;C__uncultured;O__uncultured Anaerolineaceae bacterium;F__;G__** | 0.0 | 0.0 | 0.1 | 0.0 | 0.0 | 0.0 | 0.1 |
| **P__Chloroflexi;C__uncultured;O__uncultured bacterium;F__;G__** | 0.0 | 0.0 | 0.1 | 0.2 | 0.1 | 0.0 | 1.2 |
| **P__Chloroflexi;C__uncultured;O__uncultured Chloroflexi bacterium;F__;G__** | 0.0 | 0.0 | 0.0 | 0.0 | 0.0 | 0.0 | 0.0 |
| **P__Chloroflexi;C__uncultured;Other;Other;Other** | 0.0 | 0.0 | 0.0 | 0.0 | 0.0 | 0.0 | 0.0 |
| **P__Chloroflexi;Other;Other;Other;Other** | 0.0 | 0.0 | 0.0 | 0.0 | 0.0 | 0.0 | 0.0 |
| **Sum** | **0.0** | **0.7** | **1.0** | **1.8** | **1.4** | **2.9** | **5.1** |

| 1. **Chlorobi - #OTU ID** | **DPS2** | **DPW1** | **DPW2** | **DPW6** | **DPW7** | **DPW8** | **BG** |
| --- | --- | --- | --- | --- | --- | --- | --- |
| **P__Chlorobi;C__Chlorobia;O__Chlorobiales;F__OPB56;G__uncultured bacterium** | 0.0 | 0.0 | 0.0 | 0.1 | 0.1 | 0.0 | 0.0 |
| **P__Chlorobi;C__Chlorobia;O__Chlorobiales;F__OPB56;Other** | 0.0 | 0.0 | 0.0 | 0.0 | 0.0 | 0.0 | 0.1 |
| **P__Chlorobi;C__Chlorobia;O__Chlorobiales;F__SJA-28;G__uncultured bacterium** | 0.0 | 0.0 | 0.0 | 0.0 | 0.0 | 0.0 | 0.0 |
| **P__Chlorobi;C__Chlorobia;O__Chlorobiales;F__SJA-28;G__uncultured Chlorobi bacterium** | 0.0 | 0.0 | 0.0 | 0.0 | 0.0 | 0.0 | 0.0 |
| **P__Chlorobi;C__Ignavibacteria;O__Ignavibacteriales;F__BSN166;G__uncultured bacterium** | 0.0 | 0.0 | 0.0 | 0.0 | 0.0 | 0.0 | 0.0 |
| **P__Chlorobi;C__Ignavibacteria;O__Ignavibacteriales;F__BSV26;G__uncultured bacterium** | 0.0 | 0.0 | 0.2 | 0.1 | 0.0 | 0.0 | 0.1 |
| **P__Chlorobi;C__Ignavibacteria;O__Ignavibacteriales;F__BSV26;Other** | 0.0 | 0.0 | 0.0 | 0.0 | 0.0 | 0.0 | 0.0 |
| **P__Chlorobi;C__Ignavibacteria;O__Ignavibacteriales;F__BSV40;G__uncultured bacterium** | 0.0 | 0.0 | 0.0 | 0.0 | 0.0 | 0.4 | 2.2 |
| **P__Chlorobi;C__Ignavibacteria;O__Ignavibacteriales;F__LD-RB-34;G__uncultured bacterium** | 0.0 | 0.0 | 0.0 | 0.0 | 0.0 | 1.9 | 0.0 |
| **P__Chlorobi;C__Ignavibacteria;O__Ignavibacteriales;F__PHOS-HE36;G__uncultured bacterium** | 0.0 | 0.0 | 0.0 | 0.0 | 0.0 | 0.9 | 0.0 |
| **P__Chlorobi;C__Ignavibacteria;O__Ignavibacteriales;F__PHOS-HE36;Other** | 0.0 | 0.0 | 0.0 | 0.0 | 0.0 | 0.0 | 0.0 |
| **P__Chlorobi;C__Ignavibacteria;O__Ignavibacteriales;F__SR-FBR-L83;G__uncultured bacterium** | 0.0 | 0.0 | 0.0 | 0.0 | 0.0 | 0.3 | 0.0 |
| **P__Chlorobi;C__Ignavibacteria;O__Ignavibacteriales;F__uncultured bacterium;G__** | 0.0 | 0.0 | 0.6 | 0.0 | 0.0 | 0.0 | 0.0 |
| **P__Chlorobi;C__Ignavibacteria;O__Ignavibacteriales;Other;Other** | 0.0 | 0.0 | 0.0 | 0.0 | 0.0 | 0.0 | 0.0 |
| **Sum** | **0.0** | **0.1** | **0.8** | **0.3** | **0.1** | **3.5** | **2.4** |

| 1. ***Comamonadaceae* family (Proteobacteria) - #OTU ID** | **DPS2** | **DPW1** | **DPW2** | **DPW6** | **DPW7** | **DPW8** | **BG** |
| --- | --- | --- | --- | --- | --- | --- | --- |
| **P__Proteobacteria;C__Betaproteobacteria;O__Burkholderiales;F__Comamonadaceae;G__Acidovorax** | 0.1 | 0.2 | 0.2 | 0.0 | 0.1 | 0.1 | 0.2 |
| **P__Proteobacteria;C__Betaproteobacteria;O__Burkholderiales;F__Comamonadaceae;G__Albidiferax** | 0.1 | 0.0 | 0.0 | 0.0 | 0.0 | 0.0 | 0.0 |
| **P__Proteobacteria;C__Betaproteobacteria;O__Burkholderiales;F__Comamonadaceae;G__Alicycliphilus** | 0.1 | 0.0 | 0.0 | 0.0 | 0.0 | 0.0 | 0.0 |
| **P__Proteobacteria;C__Betaproteobacteria;O__Burkholderiales;F__Comamonadaceae;G__Aquabacterium** | 0.0 | 0.0 | 0.3 | 0.1 | 0.0 | 0.0 | 0.0 |
| **P__Proteobacteria;C__Betaproteobacteria;O__Burkholderiales;F__Comamonadaceae;G__Aquincola** | 0.0 | 0.6 | 0.0 | 0.0 | 0.0 | 0.0 | 0.0 |
| **P__Proteobacteria;C__Betaproteobacteria;O__Burkholderiales;F__Comamonadaceae;G__Brachymonas** | 0.0 | 0.0 | 0.0 | 0.0 | 0.0 | 0.0 | 0.0 |
| **P__Proteobacteria;C__Betaproteobacteria;O__Burkholderiales;F__Comamonadaceae;G__Chlorochromatium** | 0.0 | 0.0 | 0.0 | 0.0 | 0.0 | 0.0 | 0.0 |
| **P__Proteobacteria;C__Betaproteobacteria;O__Burkholderiales;F__Comamonadaceae;G__Comamonas** | 0.0 | 0.0 | 0.0 | 0.0 | 0.0 | 0.1 | 0.0 |
| **P__Proteobacteria;C__Betaproteobacteria;O__Burkholderiales;F__Comamonadaceae;G__Curvibacter** | 0.1 | 0.0 | 0.0 | 0.0 | 0.1 | 0.0 | 0.0 |
| **P__Proteobacteria;C__Betaproteobacteria;O__Burkholderiales;F__Comamonadaceae;G__Delftia** | 0.0 | 0.0 | 1.9 | 0.0 | 0.0 | 0.3 | 1.3 |
| **P__Proteobacteria;C__Betaproteobacteria;O__Burkholderiales;F__Comamonadaceae;G__Hydrogenophaga** | 0.0 | 0.0 | 0.0 | 0.0 | 0.1 | 0.0 | 0.1 |
| **P__Proteobacteria;C__Betaproteobacteria;O__Burkholderiales;F__Comamonadaceae;G__Leptothrix** | 0.0 | 0.0 | 0.2 | 0.0 | 0.0 | 0.0 | 0.0 |
| **P__Proteobacteria;C__Betaproteobacteria;O__Burkholderiales;F__Comamonadaceae;G__Limnohabitans** | 0.0 | 0.0 | 0.0 | 0.0 | 0.0 | 0.0 | 0.0 |
| **P__Proteobacteria;C__Betaproteobacteria;O__Burkholderiales;F__Comamonadaceae;G__Paucibacter** | 0.0 | 0.0 | 0.0 | 0.0 | 0.1 | 0.0 | 0.0 |
| **P__Proteobacteria;C__Betaproteobacteria;O__Burkholderiales;F__Comamonadaceae;G__Polaromonas** | 0.0 | 0.0 | 0.0 | 0.0 | 0.0 | 0.0 | 0.0 |
| **P__Proteobacteria;C__Betaproteobacteria;O__Burkholderiales;F__Comamonadaceae;G__Pseudorhodoferax** | 0.0 | 0.0 | 0.0 | 0.0 | 0.0 | 0.0 | 0.0 |
| **P__Proteobacteria;C__Betaproteobacteria;O__Burkholderiales;F__Comamonadaceae;G__Rhizobacter** | 0.2 | 0.2 | 5.0 | 0.0 | 0.0 | 0.0 | 0.0 |
| **P__Proteobacteria;C__Betaproteobacteria;O__Burkholderiales;F__Comamonadaceae;G__Roseateles** | 0.0 | 0.0 | 0.0 | 0.0 | 0.0 | 0.0 | 0.0 |
| **P__Proteobacteria;C__Betaproteobacteria;O__Burkholderiales;F__Comamonadaceae;G__Schlegelella** | 0.0 | 0.0 | 0.0 | 0.0 | 0.0 | 0.0 | 0.0 |
| **P__Proteobacteria;C__Betaproteobacteria;O__Burkholderiales;F__Comamonadaceae;G__Simplicispira** | 5.6 | 6.0 | 1.5 | 0.0 | 0.0 | 0.0 | 0.9 |
| **P__Proteobacteria;C__Betaproteobacteria;O__Burkholderiales;F__Comamonadaceae;G__Sphaerotilus** | 0.0 | 0.0 | 0.0 | 0.0 | 0.0 | 0.0 | 0.0 |
| **P__Proteobacteria;C__Betaproteobacteria;O__Burkholderiales;F__Comamonadaceae;G__uncultured** | 3.8 | 0.6 | 0.2 | 0.2 | 0.5 | 0.0 | 0.5 |
| **P__Proteobacteria;C__Betaproteobacteria;O__Burkholderiales;F__Comamonadaceae;G__Variovorax** | 0.5 | 0.1 | 0.1 | 0.3 | 0.0 | 0.0 | 0.2 |
| **P__Proteobacteria;C__Betaproteobacteria;O__Burkholderiales;F__Comamonadaceae;Other** | 4.3 | 0.9 | 0.4 | 0.6 | 0.3 | 0.5 | 0.8 |
| **Sum** | **15.0** | **8.7** | **9.6** | **1.4** | **1.3** | **0.9** | **4.2** |
