# Supplementary material for: Geochemical Influence on Microbial Communities at CO2-Leakage Analog Sites
Source: Front Microbiol. 2017 Nov 9;8:2203. doi: 10.3389/fmicb.2017.02203 (PMC5684959; doi:10.3389/fmicb.2017.02203)
Supplement: Supplementary file 7 [file Table7.DOCX]

S7 Table. Top 16 archaeal genera used for NMDS plot

| **No.** | **#OTU ID** | **DPS2** | **DPW1** | **DPW2** | **DPW6** | **DPW7** | **DPW8** | **BG** |
| --- | --- | --- | --- | --- | --- | --- | --- | --- |
| 1 | **P__Euryarchaeota;C__Methanomicrobia;O__Methanosarcinales;F__Methanosarcinaceae;G__Methanosarcina** | 0 | 0 | 0 | 3.8 | 0 | 0 | **50.0** |
| 2 | **P__Euryarchaeota;C__Methanomicrobia;O__Methanosarcinales;F__Methanotrichaceae;G__Methanothrix** | 0 | 0 | 0 | 0 | 0 | 0 | **13.5** |
| 3 | **P__Euryarchaeota;C__Methanomicrobia;O__Methanosarcinales;F__unclassified_Methanosarcinales;G__** | 0 | 0 | 0 | 0 | 0 | 0 | 1.9 |
| 4 | **P__Euryarchaeota;C__Methanomicrobia;O__unclassified_"Methanomicrobia";F__;G__** | 0 | 0 | 0 | 0 | 0 | 0 | 1.9 |
| 5 | **P__Euryarchaeota;C__Methanobacteria;O__Methanobacteriales;F__Methanobacteriaceae;G__Methanobacterium** | 0 | 0 | 0 | 0 | 0 | **93.8** | 0 |
| 6 | **P__Euryarchaeota;C__Thermoplasmata;O__Methanomassiliicoccales;F__Methanomassiliicoccaceae;G__Methanomassiliicoccus** | 0 | 0 | 0 | 3.8 | 1.7 | 0 | **11.5** |
| 7 | **P__Euryarchaeota;C__unclassified_"Euryarchaeota";O__;F__;G__** | 0 | 0 | 0 | 0 | 0 | 2.1 | 0 |
| 8 | **P__Thaumarchaeota;C__Nitrososphaeria;O__Nitrososphaerales;F__Nitrososphaeraceae;G__Nitrososphaera** | **13.3** | **41.2** | **73.5** | 1.9 | **6.9** | 0 | 0 |
| 9 | **P__Thaumarchaeota;C__incertae sedis;O__Nitrosopumilales;F__Nitrosopumilaceae;G__Nitrosopumilus** | **22.2** | **17.6** | 2.0 | **71.2** | **67.2** | 0 | 0 |
| 10 | **P__Thaumarchaeota;C__unclassified_"Thaumarchaeota";O__;F__;G__** | **37.8** | **21.6** | 0 | 5.8 | **13.8** | 0 | 0 |
| 11 | **P__Diapherotrites;C__unclassified_Diapherotrites;O__;F__;G__** | 0 | 0 | 0 | 1.9 | 0 | 0 | 1.9 |
| 12 | **P__Woesearchaeota;C__Woesearchaeota Incertae Sedis AR18;O__;F__;G__** | 0 | 0 | 0 | 1.9 | 0 | 0 | 0 |
| 13 | **P__Woesearchaeota;C__Woesearchaeota Incertae Sedis AR16;O__;F__;G__** | 0 | 0 | 0 | 3.8 | 0 | 0 | 0 |
| 14 | **P__Woesearchaeota;C__unclassified_Woesearchaeota;O__;F__;G__** | 0 | 0 | 0 | 1.9 | 3.4 | 0 | 0 |
| 15 | **P__Crenarchaeota;C__Thermoprotei;O__unclassified_Thermoprotei;F__;G__** | **22.2** | 5.9 | 2.0 | 0 | 0 | 0 | **11.5** |
| 16 | **unclassified archaea;P__;C__;O__;F__;G__** | 4.4 | **13.7** | **22.4** | 3.8 | **6.9** | 4.2 | **7.7** |
