# Supplementary material for: Geochemical Influence on Microbial Communities at CO2-Leakage Analog Sites
Source: Front Microbiol. 2017 Nov 9;8:2203. doi: 10.3389/fmicb.2017.02203 (PMC5684959; doi:10.3389/fmicb.2017.02203)
Supplement: Supplementary file 8 [file Table8.DOCX]

S8 Table. Archaeal community compositions of clone library

| **Phylum** | **Class** | | **Order** | **Family** | **Genus** | **% of clones** | | | | | | |
| --- | --- | --- | --- | --- | --- | --- | --- | --- | --- | --- | --- | --- |
|  |  |  |  |  |  | **DPS-2** | **DPW-1** | **DPW-2** | **DPW-6** | **DPW-7** | **DPW-8** | **BG** |
| Euryarchaeota | Methanomicrobia | | Methanosarcinales | *Methanosarcinaceae* | *Methanosarcina* |  |  |  | 3.8 |  |  | 50.0 |
|  |  |  |  | *Methanotrichaceae* | *Methanothrix* |  |  |  |  |  |  | 13.5 |
|  |  |  |  | Unclass. *Methanosarcinales* | Unclass. *Methanosarcinales* |  |  |  |  |  |  | 1.9 |
|  |  |  | Unclass. Methanomicrobia |  | Unclass. Methanomicrobia |  |  |  |  |  |  | 1.9 |
|  | Methanobacteria | | Methanobacteriales | *Methanobacteriaceae* | *Methanobacterium* |  |  |  |  |  | 93.8 |  |
|  | Thermoplasmata | | Methanomassiliicoccales | *Methanomassiliicoccaceae* | *Methanomassiliicoccus* |  |  |  | 3.8 | 1.7 |  | 11.5 |
|  | Unclass. Euryarchaeota | |  |  | Unclass. Euryarchaeota |  |  |  |  |  | 2.1 |  |
| Thaumarchaeota | Nitrososphaeria | | Nitrososphaerales | *Nitrososphaeraceae* | *Nitrososphaera* | 13.3 | 41.2 | 73.5 | 1.9 | 6.9 |  |  |
|  | incertae sedis | | Nitrosopumilales | *Nitrosopumilaceae* | *Nitrosopumilus* | 22.2 | 17.6 | 2.0 | 71.2 | 67.2 |  |  |
|  | Unclass. Thaumarchaeota | |  |  | Unclass. Thaumarchaeota | 37.8 | 21.6 |  | 5.8 | 13.8 |  |  |
| Diapherotrites | Unclass. Diapherotrites | |  |  | Unclass. Diapherotrites |  |  |  | 1.9 |  |  | 1.9 |
| Woesearchaeota |  | |  |  | Woesearchaeota Incertae Sedis AR18 |  |  |  | 1.9 |  |  |  |
|  |  | |  |  | Woesearchaeota Incertae Sedis AR16 |  |  |  | 3.8 |  |  |  |
|  |  | |  |  | Unclass. Woesearchaeota |  |  |  | 1.9 | 3.4 |  |  |
| Crenarchaeota | Thermoprotei | | Unclass. Thermoprotei | Unclass. Thermoprotei | Unclass. Thermoprotei | 22.2 | 5.9 | 2.0 |  |  |  | 11.5 |
| Unclass. archaea |  |  | |  | Unclass. archaea | 4.4 | 13.7 | 22.4 | 3.8 | 6.9 | 4.2 | 7.7 |
| **Sum** | | | | | | 100 | 100 | 100 | 100 | 100 | 100 | 100 |
